# Analysis of *MT-ATP8* gene variants reported in patients by modeling in silico and in yeast model organism

**DOI:** 10.1038/s41598-023-36637-9

**Published:** 2023-06-20

**Authors:** Chiranjit Panja, Katarzyna Niedzwiecka, Emilia Baranowska, Jaroslaw Poznanski, Roza Kucharczyk

**Affiliations:** Institute of Biochemistry and Biophysics, Polish Academy of Sciences, Warsaw, Poland

**Keywords:** Biochemistry, Molecular biology

## Abstract

Defects in ATP synthase functioning due to the substitutions in its two mitochondrially encoded subunits *a* and *8* lead to untreatable mitochondrial diseases. Defining the character of variants in genes encoding these subunits is challenging due to their low frequency, heteroplasmy of mitochondrial DNA in patients’ cells and polymorphisms of mitochondrial genome. We successfully used yeast *S. cerevisiae* as a model to study the effects of variants in *MT-ATP6* gene and our research led to understand how eight amino acid residues substitutions impact the proton translocation through the channel formed by subunit *a* and *c*-ring of ATP synthase at the molecular level. Here we applied this approach to study the effects of the m.8403T>C variant in *MT-ATP8* gene. The biochemical data from yeast mitochondria indicate that equivalent mutation is not detrimental for the yeast enzyme functioning. The structural analysis of substitutions in subunit *8* introduced by m.8403T>C and five other variants in *MT-ATP8* provides indications about the role of subunit *8* in the membrane domain of ATP synthase and potential structural consequences of substitutions in this subunit.

## Introduction

Mitochondrial diseases are a wide group of metabolic and neuromuscular diseases, often with mental disorders, most of which are caused by defects in the functioning of oxidative phosphorylation system (OXPHOS) and a deficit in the energy-rich ATP molecule^1,2^. OXPHOS is formed by five multi-subunit complexes built from around 90 subunits of dual genetic origin, nuclear and mitochondrial^3^. In humans, mitochondrial genome (mtDNA) encodes only 13 OXPHOS subunits, but mutations in these genes account for 7–15% of patients with respiratory chain deficiency (depending on the source^4,5^). This is due to the fact that mtDNA is replicated more often than the nuclear genome, in the organelle, which is also the source of harmful reactive oxygen species^6^. The mtDNA occurs in the mammalian cell in thousands of copies, which on the one hand is advantageous because the negative effects of accumulated variants may be dominated by the wild-type mtDNA molecules. However, mtDNA is inherited randomly from the mother and pathogenic variants may be at high heteroplasmy in progeny^7,8^. When the level of heteroplasmy overcomes the critical threshold, different for each variant and depending on tissue, the disease phenotype appears^9^. Studies on cell lines and tissues from patients with heteroplasmic variants allowed to define the threshold for different variants^10and references therein^. Patient cell lines are often used for production of homoplasmic cybrid cell lines, a reliable model for the evaluation of pathogenic effect of mtDNA variants. However the nuclear background of ρ^0^ cell line generated for create cybrid, often being the tumor cell line, and fact that to deplete the cell line from mtDNA the ethidium bromide is used, contribute to conflicting finding^11^. Recently developed methods of mtDNA editing in cell lines will allow the creation of cellular models carrying particular mtDNA variants, but they are limited to specific changes^12–14^. For these reasons, different model organisms are used for research and unicellular organism *Saccharomyces cerevisiae* is an ideal model due to possibility to introduce mutations into its always homoplasmic mtDNA in defined nuclear background and ability to survive on fermentative medium when mutations disrupt the functioning of the OXPHOS^15^. The effect of mtDNA variants is studied in this model at the level of the whole organism, not the particular cell line or tissue type.

In our previous studies, we used yeast to determine the nature of *MT-ATP6* gene variants and define their mechanisms of pathogenesis^16–26^. This gene codes for the inner mitochondrial membrane located subunit *a*/ATP6 of the fifth OXPHOS complex—ATP synthase—providing the cell in ATP. Subunit *a* is directly involved in proton transport through the enzyme F_O_ domain (the membrane embedded part, while the catalytic matrix oriented part is called F1)^27–29^. In this work, we focused on *MT-ATP8* gene, which encodes the subunit *8/*ATP8 not directly involved in the proton transport, adjacent to the *a* subunit. The *MT-ATP8* and *MT-ATP6* genes show a 46 nucleotide overlap^10^. We limited our analysis to variants in the *MT-ATP8* gene fragment specific to subunit *8* only, excluding variants in a region common to both genes. To date, nine variants in this region have been described in the literature in patients with mitochondrial diseases (listed in Table 1), but due to the increasing use of NGS sequencing in diagnosis, the number of reported cases is increasing^30–33^. In the MITOMAP and ClinVar databases, these variants have the status “reported” and mostly uncertain significance. The subunit *8* primary sequence is not highly conserved, even between higher organisms. Only the beginning of yeast *8* subunit sequence shows great similarity to the human homologue. Its role in enzyme functioning is not well understood and there is no experimental work that investigated the activity and stability of ATP synthase with substitutions in this subunit. Basing on the structure of the F_O_ domain it was proposed that subunit *8* serves to stabilize the positioning of subunit *a*^34^. Thanks to the complete structures of ATP synthases from different organisms now available^28,35^, we were able to compare the structures of mammalian and yeast subunits *8* in the context of the whole holoenzyme. And although the primary sequence is different, the structure of the membrane part of subunits *8* is preserved which allows for modeling substitutions in this region. We successfully introduced the mutation equivalent to the m.8403T>C into the yeast *ATP8* gene and studied its effects in vivo and in vitro. We analyzed in silico the remaining amino acid substitutions in subunit *8* using the structure of the “humanized” bovine-derived F_O_ domain, within which the sequence of subunit *8* was replaced by the *Homo sapiens* one and their consequences for the functioning of the enzyme were proposed. So far it is the first work aiming to define the consequences of *MT-ATP8* gene variant in yeast model.

**Table 1:** *MT-ATP8* gene variants described in patients, their status in MITOMAP and ClinVar databases, amino acid substitutions in human subunit *8* and associated diseases.

mtDNA variant, m	Nr of cases	ATP8 a.a. change	Disease(s) syndrome(s)	Pathogenic score*	Status: MITOMAP/ClinVar	References
8381A>G	2	T_6_A	MIDD/LVNC cardiomyopathy-associated	0.47	Reported/bening	^45,46^
8382C>T	1	T_6_I	Episodic paralysis	0.58	Reported/uncertain significance	^33^
8403T>C	1	I_13_T	Episodic weakness and progressive neuropathy	0.77	Reported/uncertain significance	^33,43^
8411A>G	1	M_16_V	Severe mitochondrial disorder	0.63	Reported/uncertain significance	^44^
8418T>C	1	L_18_P	Severe bilateral optic neuropathy	0.73	Reported/likely pathogenic	^47^
8424T>C	1	L_20_P	Mitochondrial disease	0.85	Reported/absent	^33^
8481C>T	1	P_39_L	Tetralogy of Fallot	0.49	Reported/uncertain significance	^66^
8490T>C	1	M_42_T	Peripheral neuropathy of T2DM, multiple sclerosis	0.36	Reported/Bening	^30,67^
8447delA	1	L_29_Ter	Acute lymphoblastic leukemia	–		^68^

*LVNC* Left Ventricular Non-Compaction: Mid-myocardial Distribution, *MIDD* Maternally-Inherited Diabetes and Deafness. *> 0.7 means high pathogenicity score^42^.

## Results

### Structural consequences of subunit *8* substitutions in the ATP synthase F_O_ domain

**Figure 1:**
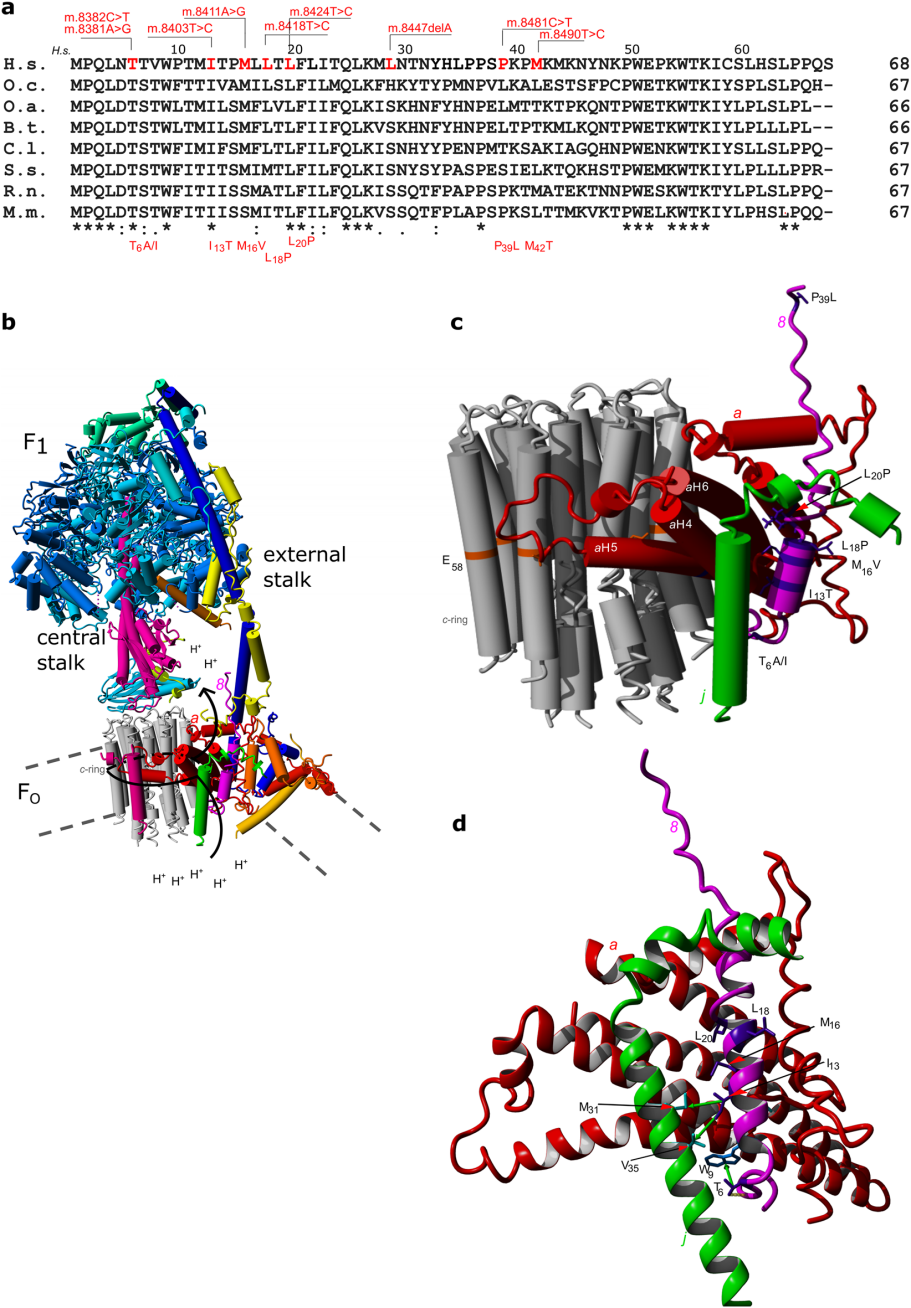
Conservation of subunits *8* and localization of the residues substituted due to variants in patients mtDNA. (**a**) Sequence alignment of subunit *8* from vertebrates. The abbreviations mean *H.s.*—*Homo sapiens, B.t.*—*Bos taurus*, *S.s.*—*Sus scrofa*, *O.c.*—*Oryctolagus cuniculus*, *O.a.*—*Ovis aries*, *C.l.*—*Canis lupus, R.n.*—*Rattus norvegicus, M.m.*—*Mus musculus.* The *H.s.* amino acids numbering is above the alignment. The mtDNA variants and subunit *8* substitutions are indicated. (**b**) Overall view of the mammalian ATP synthase monomer. Each subunit is marked by different color. The membrane is indicated by dotted line and the pathway along which protons move from the intermembrane space to the mitochondrial matrix is indicated by the arrow. (**c**) View from membrane of the mammalian F_O_ domain subunits *c*, *a*, *8* and *j*. The amino acid residues *c*E_58_ directly involved in the proton translocation are shown as an orange belts. The positions of the substitutions in subunit *8* are indicated by deep violet belts and sticks. (**d**) View of the *a* (red), *8* (magenta) and *j* (green) subunits interface. Five side chains substituted in patients (*8*T_6_, *8*I_13_, *8*M_16_, *8*L_18_ and *8*L_20_) are drawn as deep violet sticks. The hydrophobic interactions are indicated as green arrows, the residues *j*M_31_ and *j*V_35_ are indicated by cyan sticks. *8*W_9_ is colored in blue. The internal hydrogen bond of *8*T_6_ side-chain oxygen with the backbone carbonyl group of *8*L_4_ is indicated by yellow dotted line. The panels b, c and d were prepared with the Yasara Structure package (version 21.8.27, licensed to J.P.; http://www.yasara.org/)^65^, combining 6cp5 and 6zbb PDB records.

Nine variants in the *MT-ATP8* gene fragment specific for subunit *8* were described in patients suffering from mitochondrial diseases in the literature. Their positions in mtDNA, status in the MITOMAP and ClinVar databases, substitutions in subunit *8*, associated diseases, pathogenicity score, and references are given in Table 1 (see also Fig. 1a,c). ATP synthase is a motor enzyme located in the inner mitochondrial membrane, built from the membrane-embedded F_O_ and matrix exposed F_1_ domains, connected by the central and external stalks (Fig. 1b). During catalysis the ring of subunits *c* rotates together with the central stalk, while the catalytic hexamer of α/β subunits (F_1_ domain) and the stator does not rotate (Fig. 1b). The γ subunit of the central stalk protrudes into the F_1_ domain, causing conformational changes favoring substrates’ binding, ATP synthesis, and release^36^. Rotation of the *c*-ring is coupled to the proton transport through the channel formed between the *c*-ring and tightly adjusted subunit *a*. Subunit *8* is located in the membrane part of the ATP synthase stator and is tightly adjusted to subunit *a* and *i/j*, forms an α-helix, spanning the membrane and protrudes into the matrix (Fig. 1b–d). Subunit *8* is not involved in the catalytic proton transfer because it is remote from the *c*-ring. Due to the significant differences in the sequence of yeast and human subunit *8*, the application of yeast model to study the effects of mutations equivalent to human *MT-ATP8* variants on enzyme function is limited. For this reason, we first used the available bovine ATP synthase structure to analyze what structural consequences the particular substitution in subunit *8* described in patients has.

In bovine and porcine ATP synthase solely the structure of the first 29 residues of subunits *8* was solved and the remaining C-terminal region was missing^28,37^. Bearing in mind the identity of the two mammalian structures of the N-terminal fragment of subunit *8*, we decided to analyze in silico “humanized” bovine F_O_ domain, in which that of *Homo sapiens* replaced the sequence of bovine subunit *8*. We used two classes of descriptors to quantify the thermodynamic effects of a particular residue replacement at a given position: total free energy change of the stability of the subunit *8* peptide and the stabilization/destabilization effect on the pairwise inter-subunits interactions.

The first six residues of subunit *8* helix are bent ninety degrees towards subunit *a* helix 4 (*a*H4, where *a* indicates the subunit, H means helix). The conserved threonine in position 6 causes the *8*-helix to fold towards the *a* subunit. The internal hydrogen bond of its side-chain oxygen with the backbone carbonyl group of leucine in position 4 (*8*L_4_) stabilizes subunit *8* backbone bending (Fig. 1d, yellow dotted line). The neighboring tryptophan *8*W_9_ interacts with *a*L_98_ and *a*S_99_ in the helix 4 of subunit *a*, stabilizing its positioning. The variants m.8381A>G and m.8382C>T led to substitution of threonine in position 6 to alanine or isoleucine (*8*T_6_A/I), respectively. Replacement of threonine by alanine moderately destabilizes the subunit *8* bending (ΔΔG_fold_ = 0.6 kcal/mol), while isoleucine in this position has the opposite effect (ΔΔG_fold_ = − 0.7 kcal/mol) and moreover it interacts with the *8*W_9_ and makes this fragment more compact (Fig. 1d, green arrow). In both cases, the positioning of subunit *a* may be changed, which may minutely affect the proton channel functioning.

The m.8403T>C introduces threonine in the place of isoleucine in position 13 of subunit *8* (*8*I_13_T). *8*I_13_ interacts with subunit *j* residues *j*V_35_ and *j*M_31_, forming the hydrophobic cluster (Fig. 1d, green arrows). Its side-chain is also involved in Van der Waals interactions with subunit *a* residues *a*G_103_ and *a*S_99_, which stabilize the positioning of subunit *a* relative to subunit *8*. Threonine in this position breaks these interactions pattern (ΔΔG_fold_ = 3.2 kcal/mol), which may affect the subunit *a* placement.

The m.8411A>G introduces valine in the place of methionine in position 16 of subunit *8* (*8*M_16_V). The *8*M_16_ side-chain forms the hydrophobic cluster with residues *a*M_100_, *a*G_103_, *a*M_104_, *8*M_12_, and *j*M_31_. Substitution with valine in this position substantially modifies these interactions (ΔΔG_fold_ = 3.7 kcal/mol), which may destabilize the interface between these three subunits, especially with subunit *a*, and in consequence its functioning.

The m.8418T>C and m.8424T>C led to replacement of both *8*L_18_ or *8*L_20_ by proline, which destabilizes the whole F_O_ domain (ΔΔG_fold_ = 4.0 or 10 kcal/mol, respectively). *8*L_18_ interacts with *a*T_21_ and *a*L_25_, while *8*L_20_ interacts with *a*L_75_, *a*F_78_, *a*S_74_, *a*M_71_, and *a*M_104_. Substitution with proline in one of these two positions introduces substantial steric clashes, which must be further partially compensated by a global conformational change (not modeled in FoldX). That will strongly affect the positioning of subunit *a* and, in consequence, the functioning of the channel.

The two remaining substitutions: *8*P_39_L, and *8*M_42_T (Table 1, Fig. 1a,c), are located in not defined subunit *8* fragment in two mammalian ATP synthase structures^28^, so the results must be regarded roughly qualitative. The thermodynamic effect of *8*P_39_L substitution (ΔΔG_fold_ < 1 kcal/mol) remained almost negligible.

### Consequences of the ATP8-I_13_T equivalent substitution in yeast subunit *8* (*8*L_13_T)

**Figure 2:**
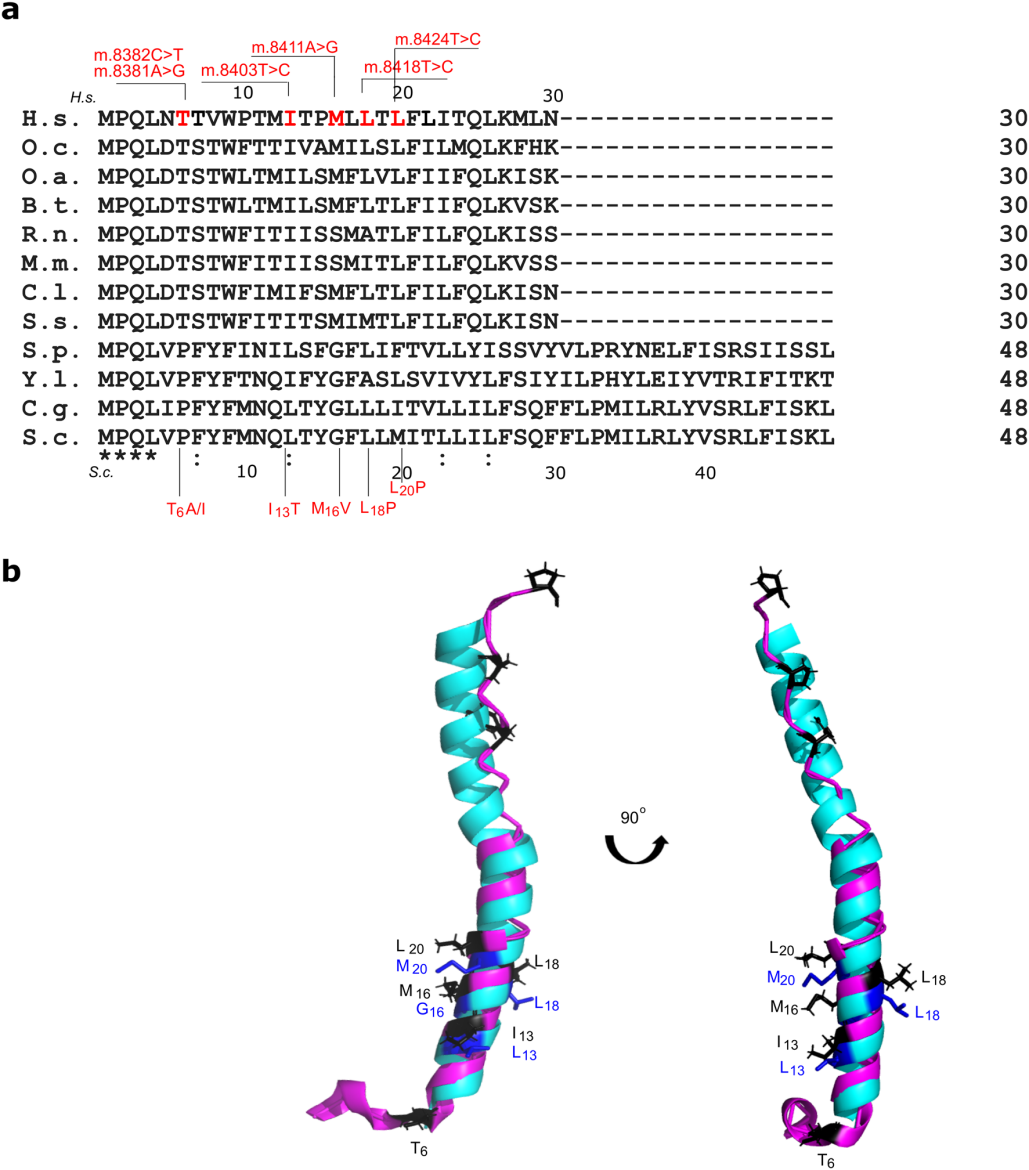
Mammalian and yeast subunits *8* overlap. (**a**) Sequence alignment of subunit *8* from vertebrates (the first 30 amino acids) and yeasts. The abbreviations mean: *S.p.*—*Schizosaccharomyces pombe*, *Y.l.*—*Yarrowia lipolytica, C.g.*—*Candida glabrata* and *Saccharomyces cerevisiae* (*S.c.*) and remaining are explained in the legend to Fig. 1. The human variants and subunit *8* substitutions are indicated above or below the alignment, respectively. (**b**) Super positioning of the subunits *8* from bovine (magenta) and yeast (cyan) (pdb codes 6zbb and 6cp5, respectively) done using the Pymol™, (version 2.3.2, licensed to R.K.; https://pymol.org/2/). The first 6 residues of the yeast and second part of the bovine (starting from 29th residue) subunits were not resolved in the published structures therefore are not visible. Positions found to be substituted in patients are indicated by black sticks while the corresponding in yeast subunit are shown in deep blue.

Yeast as a model can be used to study the effects of mutations in humans when these mutations affect conserved amino acid residues. Comparing the sequences of the human and yeast subunits *8*, a similarity is seen within the first 18 amino acid residues, where the first 4 residues and leucine in position 18 are conserved while similar residues are present in positions 9 and 13: W_9_ and I_13_ in mammals is replaced by F_9_ and L_13_ in yeast, respectively (Fig. 2a). Basing on the sequence alignment we aimed to introduce the threonine in place of leucine in position 13 and valine in place of leucine in position 18 of yeast subunit *8*, succeeding with the first substitution. With the publication of the subunit *8* structures in vertebrates and yeast, it was possible to compare them. As can be seen in Fig. 2b, the first 20 amino acid residues coincide quite closely in the structure of subunit *8* of vertebrates and yeast, validating our choice. Because subunit *8* was proposed to stabilize the subunit *a*^34^ we constructed two variants of *8*L_13_T substitution: in the wild type mtDNA (strain CPY1-WT, Table 2) and in the mtDNA in which subunit *a* was fused to HisHA tags at the C-terminus (CPY1) in the goal to pulldown the whole enzyme complex and assess its stability (see “Methods” for description of strains construction^38^).

**Table 2:** Genotypes and sources of yeast strains.

Strain	Nuclear genotype	mtDNA	Source
DFS160	*MAT****α**** leu2Δ ura3-52 ade2-101 arg8::URA3 kar1-1*	ρ^0^	(1)
NB40-3C	*MAT****a**** lys2 leu2-3,112 ura3-52 his3::HinDIII arg8::hisG*	ρ^+^ *cox2-62*	(1)
MR6	*MAT****a**** ade2-1 his3-11,15 trp1-1 leu2-3,112 ura3-1 CAN1 arg8::hisG*	ρ^+^	(2)
MR6-ATP6-HisHA	*MAT****a**** ade2-1 his3-11,15 trp1-1 leu2-3,112 ura3-1 CAN1 arg8::hisG*	ρ^+^ *ATP6-HisHA ATP8*	^38^
MR6-atp8Δ ATP6-HisHA	*MAT****a**** ade2-1 his3-11,15 trp1-1 leu2-3,112 ura3-1 CAN1 arg8::hisG*	ρ^+^ *ATP6-HisHA atp8::ARG8*^*m*^	^38^
MR6-*atp8Δ*	*MAT****α**** ade2-1 his3-11,15 trp1-1 leu2-3,112 ura3-1 CAN1 arg8::hisG*	ρ^+^ *ATP6-WT atp8::ARG8*^*m*^	^38^
RKY68	*MAT****α**** leu2Δ ura3-52 ade2-101 arg8::URA3 kar1-1*	ρ^−^ *ATP8 COX2*	This study
KNY120	*MAT****a**** ade2-1 his3-11,15 trp1-1 leu2-3,112 ura3-1 CAN1 arg8::hisG*	ρ^+^ *ATP6-WT ATP8*	This study
RKY194	*MAT****a**** ade2-1 his3-11,15 trp1-1 leu2-3,112 ura3-1 CAN1 arg8::hisG*	ρ^+^ *ATP6-WT atp8::ARG8*^*m*^	This study
CPY1synth	*MAT****a**** leu2Δ ura3-52 ade2-101 arg8::URA3 kar1-1*	ρ^−^ *atp8-L13T COX2*	This study
CPY1	*MAT****a**** ade2-1 his3-11,15 trp1-1 leu2-3,112 ura3-1 CAN1 arg8::hisG*	ρ^+^ *ATP6-HisHA atp8-L13T*	This study
CPY1-WT	*MAT****a**** ade2-1 his3-11,15 trp1-1 leu2-3,112 ura3-1 CAN1 arg8::hisG*	ρ^+^ *ATP6-WT atp8-L13T*	This study

### Respiratory growth

**Figure 3:**
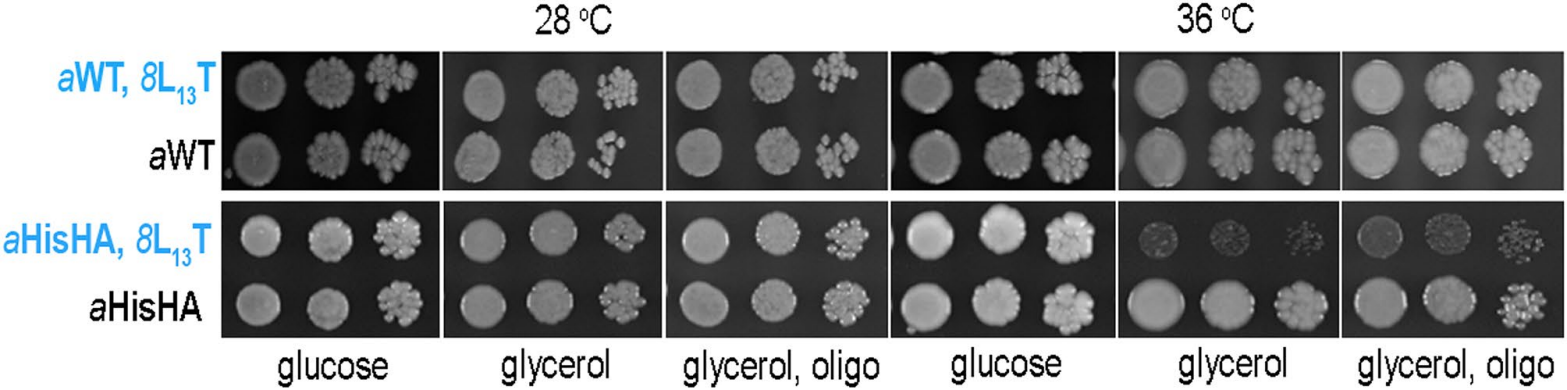
Respiratory growth phenotypes of strains bearing the *8*L_13_T in the background of *a*WT or *a*HisHA. Cells from the indicated strains grown in glucose pre-cultures were serially diluted and spotted on rich glucose or glycerol plates with or without oligomycin (0.5 μg/mL) and incubated at 28 or 36 °C. Plates without oligomycin were scanned after three days of incubation while those with the drug after four days of incubation.

The growth of *8*L_13_T cells was checked on solid and liquid fermentative (glucose) and respiratory (glycerol) media at both temperatures routinely used for yeast cells: physiological 28 °C and elevated 36 °C (conditions of the mild heat stress). While the *8*L_13_T substitution in wild type enzyme does not slow down the growth of yeast cells at both temperatures and on both types of media liquid and solid, it leads to very poor respiratory growth when coexists with subunit *a*HisHA on solid medium and slows down the growth in liquid respiratory medium at elevated temperature (Fig. 3, Fig. S1). Interestingly these cells reached lower plateau also in liquid fermentative medium, especially when grown at elevated temperature, what additionally indicates on the lower capacity to use non-fermentative carbon sources (here the ethanol produced during logarithmic growth by fermentation of glucose, Fig. S1). The good respiratory growth does not imply that the mutation has no deleterious effects on ATP synthase, because decreasing ATP synthase activity by ~ 85% is required to affect significantly growth of yeast cells on non-fermentable substrates^39^. However, the ATP production deficits lower than 85% may be manifested by the growth phenotype on respiratory medium supplemented with sub-inhibitory concentration of oligomycin—a drug that inhibits rotation of the *c*-ring—because less of this drug is needed to reach the ATP synthase threshold activity^19^. Like *8*L_13_T substitution alone does not impact the respiratory growth in the presence of oligomycin, coexistence of *8*L_13_T with *a*HisHA does it at elevated temperature (Fig. 3).

### Respiration and ATP synthase activities

To measure the efficiency of OXPHOS functioning, mitochondria were isolated from cells bearing *8*L_13_T grown in rich galactose medium at two temperatures 28 and 36 °C. Galactose is a fermentative carbon source which does not repress the expression of mitochondrial genes^40^. To reflect the activities of respiratory chain we measured the rate of oxygen consumption by mitochondria using NADH as a substrate (state 4). To reflect the activity of respiratory chain and ATP synthase the ADP was added after NADH (oxygen consumption upon ATP synthase operation, state 3). Addition of the proton ionophore CCCP before NADH let to measure the uncoupled, maximal respiration. Ascorbate/TMPD in the presence of CCCP was used to measure the maximal complex IV activity. ATP synthesis was assayed in the conditions of state 3.

**Figure 4:**
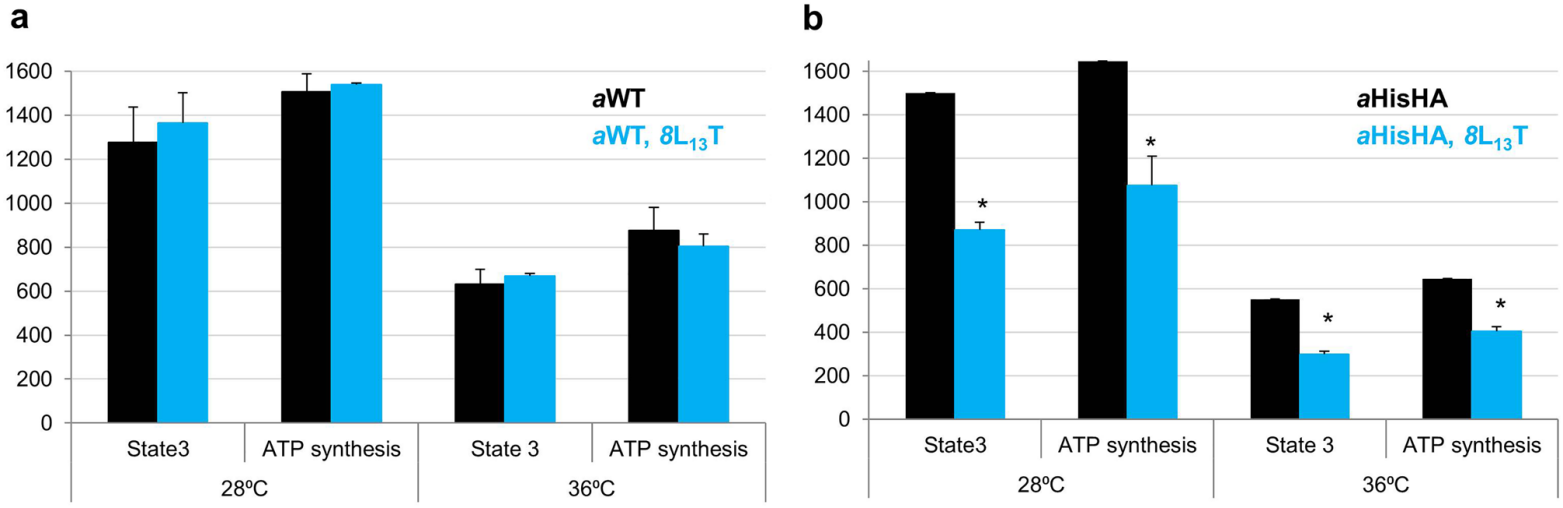
State 3 and ATP synthesis in mitochondria from *8*L_13_T *a*WT (**a**) and *8*L_13_T *a*HisHA (**b**) cells. The oxygen consumption at state 3 is given in the nmol of O_2_ min^−1^ mg^−1^ and ATP synthesis values are represented in nmol of ATP min^−1^ mg^−1^. The histograms show the data from three biological repetitions. Statistical significance is shown: *p < 0.05. The figure is relative to Tables S1 and S2.

The activities reflect the respiratory growth deficits, described above. The *8*L_13_T substitution in wild type enzyme does not slow down the oxygen consumption and ATP synthesis in mitochondria isolated from cells grown at both temperatures (Fig. 4a, Table S1). Consistently with slower respiratory growth, the oxygen consumption (state 4 and state 3) and ATP synthesis were reduced by ~ 45% and 35%, respectively, in mitochondria extracted from the *8*L_13_T *a*HisHA cells, similarly at both temperatures (Fig. 4b, Table S2). Although the state 3 and the ATP synthesis were significantly reduced, the yield in ATP per electron transferred to oxygen in mitochondria was the same as the one measured in control mitochondria. The uncoupled, maximal respiration and activity of complex IV were reduced similarly, in accordance with the adaptation of complex IV biogenesis to the proton transport activity of F_O_ (Table S2^41^). Then we evaluated the F_1_-mediated ATP hydrolysis activity on non-osmotically protected mitochondria at pH 8.4, the conditions under which this activity is maximal. A 15% reduction in this activity was observed in mitochondria from *8*L_13_T *a*HisHA cells grown at elevated temperature (Table S2).

**Figure 5:**
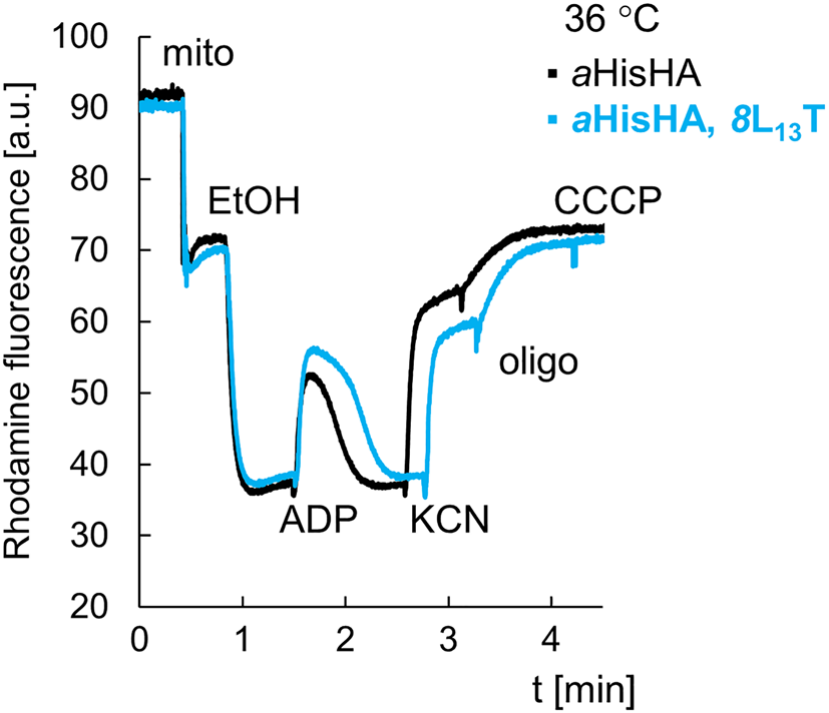
Variations in mitochondrial inner membrane potential in mitochondria from *8*L_13_T *a*HisHA cells grown at elevated temperature. The tracings show how the mitochondria responded to externally added ADP. The additions were 75 μM ADP, 0.5 μg/mL Rhodamine 123, 75 μg/mL mitochondrial proteins (Mito), 10 μL ethanol (EtOH), 2 mM KCN, 4 μg/mL oligomycin (oligo), and 4 μM CCCP. The shown tracings are representative of three biological repetitions.

To further evaluate the influence of the *8*L_13_T substitution in subunit *8* on oxidative phosphorylation we monitored changes in the mitochondrial transmembrane potential (Δψ) using a cationic dye Rhodamine123, the uptake rate of which is a highly reproducible and sensitive parameter for estimation of Δψ. Mitochondria are energized by ethanol, then ADP is added, what induce Δψ due to proton reentry through the F_1_F_O_-ATP synthase, which is normally rebuild during one minute in wild type mitochondria. Then the respiratory chain is blocked by addition of potassium cyanide (KCN) what induce the immediate drop in Δψ which is not total because the ATP synthesized during the previous step of the experiment become hydrolyzed by ATP synthase, which pumps protons to the IMS and sustains the potential. Addition of oligomycin inhibits ATP synthase what is manifested by fast drop in Δψ. The CCCP is added at the end of the experiment to normalize it, as it dissipates Δψ totally. The Δψ variations in mitochondria from *8*L_13_T cells in wild type subunit *a* were similar to those in the control mitochondria, independently on the growth temperature of cells. When the *8*L_13_T coexists with subunit *a*HisHA the changes are similar in mitochondria from cells grown at physiological temperature for yeast, but not in those from cells grown at elevated temperature. In these conditions a significantly longer time is needed to reestablish the Δψ after ADP addition, in proportion to the reduced capacity of those mitochondria to respire and synthesize ATP (Fig. 5).

### ATP synthase stability

**Figure 6:**
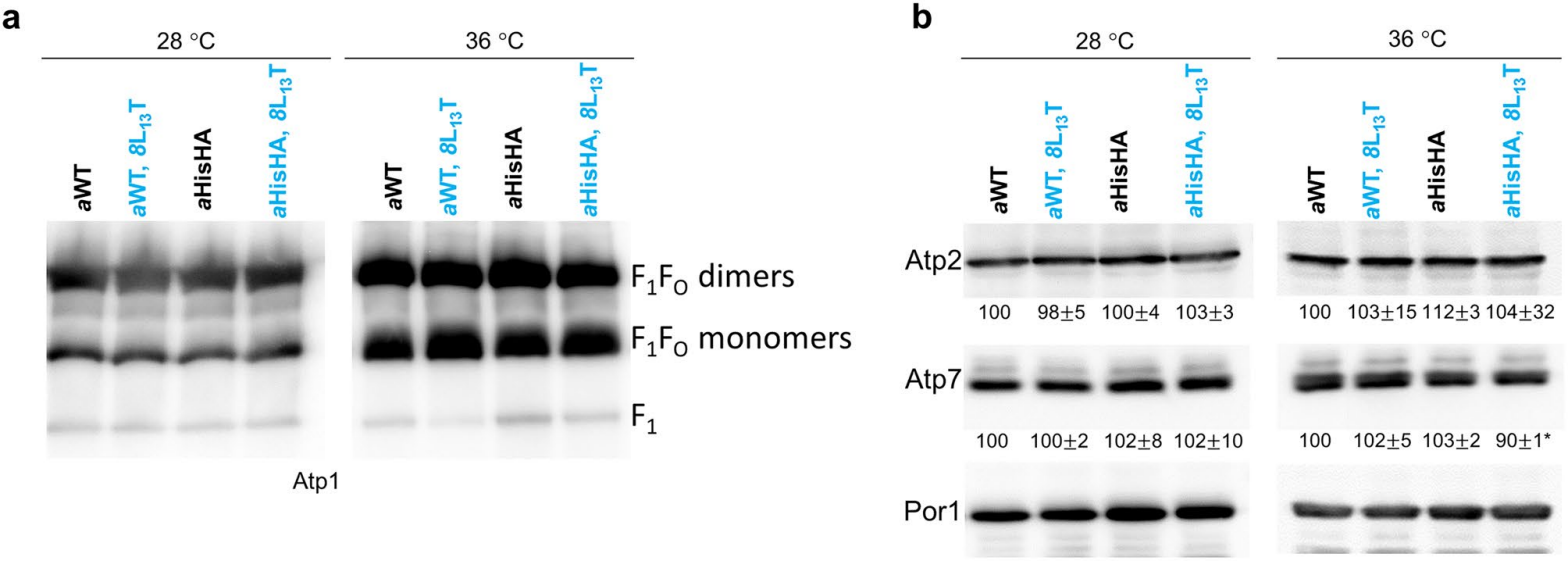
Assembly and stability of ATP synthase complexes in *8*L_13_T cells. (**a**) The ATP synthase complexes were liberated from the mitochondrial membrane by digitonin (1.5 g/g protein) and 200 μg of proteins were separated by BN-PAGE in gels containing a 3–10% polyacrylamide gradient. The proteins were transferred to a PVDF membrane and probed with antibodies against, Atp1 (subunit α). Dimeric, monomeric and free F_1_ subdomains of ATP synthase are indicated. (**b**) Total cellular protein extracts were separated by SDS-PAGE and then transferred to a nitrocellulose membrane and probed with antibodies against the indicated proteins. The intensity of bands was calculated using ImageJ and normalized to the porin used as a loading control. The standard errors and p-values were calculated from results of at least three independent experiments. Samples from the indicated strains were analyzed together on one gel transferred to the membrane and the original blots are presented in SupplementaryRowImages pages 2–5. The representative gels fragments are shown.

The stability and amount of fully assembled ATP synthase complexes were analyzed by BN- and SDS-PAGE electrophoresis followed by Western blotting. The complexes were liberated from the inner membrane of previously isolated mitochondria by the mild detergent—digitonin, the conditions under which the dimers and monomers of the enzyme are well preserved. The *8*L_13_T bearing ATP synthase complexes, independently on the subunit *a* variant, were stable and accumulated in the amount comparable to the control mitochondria (Fig. 6a). The accumulation of ATP synthase subunits in total protein extracts from cells was not decreased, indicating on no impact of the *8*L_13_T substitution on stability of the enzyme (Fig. 6b). Then we performed the pull-down of the ATP synthase complexes from the mitochondria using the Ni-NTA agarose to further study the enzyme stability. As shown on Fig. S2 the amount of ATP synthase subunits purified from mitochondria containing wild type subunit *8* and *8*L_13_T variant were similar.

## Discussion

In the mitochondrial *MT-ATP8* gene, nine variants introducing the substitutions into subunit *8* of ATP synthase have been described to date in patients with mitochondrial diseases (Table 1). The pathogenicity of any of these variants has not been confirmed (MITOMAP/ClinVar). All but one was described in single case/family. Biochemical data from patients' cells are missing, except one report in which m.8382C>T, m.8403T>C and m.8424T>C variants were studied permitting to conclude about pathogenicity of m.8424T>C^33^. Therefore, we aimed to use *S. cerevisiae*, which is an ideal model organism to study the effects of variants in the *MT-ATP6* gene, to model variants in the *MT-ATP8*. The low sequence conservation of subunit *8* limits the use of this organism as a model to study the effects of substitutions in subunit *8* (Fig. 2a) however, the structures of the membrane fragments of human and yeast subunits *8* are highly conserved in evolution. Therefore, we believe that amino acid residues introduced at the corresponding positions in the yeast and human subunit *8* will result in similar changes in the structure and functioning of the F_O_ domain. The substitution of *8*L_13_ of yeast subunit *8*, equivalent to *8*I_13_ in the human subunit, has no detrimental consequences for the functioning of the yeast ATP synthase, arguing that this variant is not pathogenic. However, the significant (of about 40%) decrease of oxygen consumption and ATP production was found in mitochondria when this substitution coexisted with the modification of the C-terminus of subunit *a* by its fusion with the six histidine residues and HA epitope sequence of nine amino acid residues (YPYDVPDYA)^38^. As individually, both these modifications do not affect the activity of ATP synthase, when combined, they reduce the efficiency of proton transport by 40% (but not the stability of the enzyme). The explanation is that each of these modifications individually can be compensated within these subunits, but when they occurred together, such compensation is not possible. The C-terminal HA tag and 6 histidine residues are at the end of subunit *a*H6, which is directly adjacent to the *c*-ring. The compensation of changes within the subunit *a* introduced by HisHA tag may be located within subunit *a* helices 6, 5 and even 4, because the *8*L_*13*_T substitution which disturbs the subunit *a*H4, prevents it. It is therefore possible that the *8*L_*13*_T variant may be harmful in certain genetic background(s). With an energy change of ΔΔG_fold_ = 3.2 kcal/mol, stability and interactions of subunit *8* with the subunit *a* neighboring residues (*a*G_103_ and *a*S_99_) may be disturbed, which may be of great importance for ATP synthase functioning. Interestingly the pathogenicity score of m.8403T>C was high^42^ and patient suffered from progressive neuropathy. The measurements performed on the patients’ primary fibroblasts showed decreased complex IV but normal ATP synthesis activity^33,43^. Further experimental studies are needed to clearly define the nature of m.8403T>C variant.

The structures of F_O_ domain of ATP synthase from many organisms allowed to understand the enzyme mechanism of action. The in silico analysis of amino acid substitutions in subunits of ATP synthase provides much information about the potential mechanisms by which they affect the enzyme functioning. In the structures of mammalian ATP synthases, recently published, the ATP8 protein was solved within its first 29 amino acid residues permitting to analyze only six substitutions^28,37^. The in silico analysis of *8*T_6_A, *8*T_6_I, *8*M_16_V, *8*L_18_P and *8*L_20_P substitutions provided important information’s about the role of these residues in subunit *8*: The *8*T_6_ is important for the shape of subunit *8*, which stabilize the placement of the subunit *a*H4, while the *8*M_16_, *8*L_18_ and *8*L_20_ are crucial for the hydrophobic interactions of subunit *8* with subunit *a* and *j*. This finding is in accordance with previously proposed role for subunit *8* in stabilization of subunit *a* in the F_O_^34^. The substitutions *8*T_6_A and *8*T_6_I minutely change the ΔΔG energy and may destroy the interactions of subunit *8* with the neighboring residues of subunit *a*. They may have slight indirect effects on the functioning of the proton channel, but experimental research is needed to define their consequences. In accordance to these finding substitutions of *8*T_6_ were associated with milder diseases phenotypes: cardiomyopathy or diabetes with deafness. The subunit *8* energy change due to *8*M_16_V was significant and this variant was associated to more severe mitochondrial disease leading to death in childhood^33,44–46^. This can be explained by the fact that *8*M_16_ is located very close to the central part of the *a* subunit, where amino acid residues directly involved in proton transport through F_O_ are located. The located close *8*L_18_P and *8*L_20_P changes are very costly energetically, proline is excluded from both these positions of subunit *8*, moreover significant respiratory chain deficiencies were found in m.8424T>C cybrids. The huge structural destabilization assigned in silico to the *8*L_20_P variant (ΔΔG_fold_ = 10 kcal/mol) may directly affect the biogenesis of subunit *8* and cause its improper assembly to ATP synthase F_O_ domain, as suggested in Ref.^33^. These findings correlate with severe diseases in patients and speak for pathogenic character of those two variants^33,47^. The super-positioning of subunits *8* from yeast and bovine indicates that both variants may be modelled in yeast model organism for experimental verification of this conclusion.

## Conclusion

In silico analysis suggests that two variants m.8418T>C and m.8424T>C may be the cause of the mitochondrial disease in the reported cases. Mutation equivalent to the m.8403T>C variant in *MT-ATP8* gene does not affect the ATP synthase functioning in *S. cerevisiae*, which indicates its non-pathogenic character. The obtained results on its two yeast models suggest that structural compensations of variants within the subunits of ATP synthase are possible. This fact, as well as our previous work based on the analysis of genetic suppressors of pathogenic variants in the *a* subunit, are an argument for designing small molecules/peptides, potential drugs, that would induce structural changes and restore the enzyme function.

## Methods

### Growth media

The media used for growing yeast were: fermentative: YPDA (1% Bacto yeast extract, 1% Bacto Peptone, 2% glucose for standard growth or 10% glucose for selection of the cytoductants, 40 mg/L adenine), YPGalA (1% Bacto yeast extract, 1% Bacto Peptone, 2% galactose, 40 mg/L adenine), respiratory: YPGlyA (1% Bacto yeast extract, 1% Bacto Peptone, 2% glycerol, 40 mg/L adenine). BIOL-Leu (5% glucose, 182.5 g/L sorbitol, 1.7 g/L Yeast Nitrogen base without amino acids, 5 g/L ammonium sulfate, 0.8 g/L CSM-Leu drop out mix, and 40 mg/L adenine) is a fermentative medium for selection of mitochondrial transformants. Media were solidified by the addition of 2% (w/v) Bacto agar. Yeast cells were grown at 28 or 36 °C, cultures in liquid media were shaken at 180 rpm. Oligomycin was added to the media at the concentration of 0.5 µg/mL. Growth curves were established with the Bioscreen CTM system.

### *ATP8* gene mutagenesis and construction of the *atp8-L13T* mutant strains

The equivalent mutation to m.8403T>C was introduced into *ATP8* gene cloned into pMOS vector^48^ with the Q5^®^ Site-directed Mutagenesis Kit of *NEB*iolabs. The sequences of primers used for mutagenesis reaction are: 5′ TATTATTAATTTTATTCTCACAAT**TC**TTTTTACCTATG 3′ (the changed bases are indicated in bold) and 5′ GAATCATTAATAAGAAACCATATGTTGTTTGATTCATAAAATAAAATGGAAC 3′. The DNA fragment containing the *atp8-L13T* was liberated by XbaI-NdeI digestion from pMOS-ATP8-based plasmids resulting from mutagenesis reaction and ligated at the same sites with pJM2 giving the pCP1 plasmid^49^. The pJM2 contains the yeast mitochondrial *COX2* gene, which serves for identification of mitochondrial transformants.

The mutations into mtDNA are introduced into the MR6 strain^50^. It is a derivative of W303-1B strain in which the nuclear *ARG8* gene was replaced with *HIS3*, contains mitochondrial genome of S288C strain that has been entirely sequenced, and has a wild-type *CAN1* gene, encoding a basic amino acid permease. The RKY194 strain was obtained by the change of the mating type of MR6-atp8Δ *MAT****α*** strain (*atp8Δ-ATP6-WT*, provided by A. Tzagoloff) using a plasmid encoding the HO endonuclease^51^. The isogenic wild type control strain for RKY194 was constructed by crossing RKY194 with the RKY68, the ρ^*−*^ synthetic bearing in the mtDNA only wild type *ATP8* and *COX2* genes, and selection the respiring progeny bearing the nucleus of RKY194. To receive the *atp8-L13T* mutant the pCP1 plasmid was introduced by co-transformation with the *LEU2* gene containing plasmid Yep351 into the ρ^0^ strain DFS160 by microprojectile bombardment using a biolistic PDS-1000/He particle delivery system (Bio-Rad) as described^15^. To learn more about the technique developed by us see^52^. Mitochondrial transformants were identified among the Leu^+^ nuclear transformants by their ability to produce respiring clones when mated to the non-respiring NB40-3C strain bearing a deletion in the mitochondrial *COX2* gene. The resulting clone ρ^*−*^ synthetic CPY1synth (Table 2) was crossed to strains MR6 atp8Δ-ATP6-HisHA (provided by prof. A. Tzagoloff) or RKY194 (*atp8Δ-ATP6-WT*). Because the ρ^*−*^ synthetic strain bears the mutation *kar1-1* which blocks the fusion of the nuclei, during the crosses the cytoplasm and the mitochondrial network fuse permitting for homologous recombination between the two mtDNA molecules and the replacement of *ARG8m* gene present in the *ATP8* locus by *atp8* sequence bearing *atp8-L13T* mutation. During the two passages of cells resulting from the crosses in the rich 10% glucose medium the mtDNAs separate till the homoplasmic state. The resulting CPY1 (in background of *ATP6*-HisHA) and CPY1-WT (in background of *ATP6*-WT) strains bearing in the complete (ρ+) mtDNA the *atp8-L13T* gene variant was identified by ability to grow on non-fermentable carbon source glycerol and inability to grow in the absence of arginine. The presence of mutation was verified by DNA sequencing of the PCR amplified *ATP8* locus with oligonucleotides oATP8-3 5′-TGTCAGTTATTTTATATTAATGTTTAATC-3′ and oATP8-4 5′-ATATATATATATAAATATATAGTCCGTAAGG-3′.

### Measurement of mitochondrial respiration, ATP synthesis/hydrolysis and membrane potential

The mitochondria were prepared from yeast cells grown in rich galactose medium (YPGalA) at 28 °C or 36 °C by the enzymatic method described in Ref.^53^. YPGalA is a fermentative medium in which the mitochondrial genes are not repressed. This growth condition reduces the probability for selection of the spontaneous suppressors in the respiratory deficient mutants and is used in our routine analysis of mitochondrial mutants. For respiration and ATP synthesis assays, mitochondria were diluted to 0.075 mg/mL in respiration buffer (10 mM Tris-maleate (pH 6.8), 0.65 M mannitol, 0.36 mM EGTA, and 5 mM Tris–phosphate). Oxygen consumption rates were measured using a Clarke electrode after consecutively adding 4 mM NADH (state 4 respiration), 150 µM ADP (state 3) or 4 µM carbonyl cyanide m-chlorophenylhydrazone (CCCP) (uncoupled respiration), as previously described^54^. The rates of ATP synthesis were determined under the same experimental conditions in the presence of 750 µM ADP; aliquots were withdrawn from the oxygraph cuvette every 15 s and the reactions were stopped with 3.5% (w/v) perchloric acid, 12.5 mM EDTA. The ATP in samples was quantified using the Kinase-Glo Max Luminescence Kinase Assay (Promega) and a Beckman Coulter's Paradigm Plate Reader. Participation of F_1_F_O_-ATP synthase to ATP production was assessed by measuring the sensitivity of ATP synthesis to oligomycin (3 μg/mL). Variations in transmembrane potential (ΔΨ) were evaluated in the respiration buffer containing 0.150 mg/mL of mitochondria and the Rhodamine 123 (0.5 μg/mL), with λexc of 485 nm and λem of 533 nm under constant stirring using a Cary Eclipse Fluorescence Spectrophotometer (Agilent Technologies, Santa Clara, CA, USA)^55^. The specific ATPase activity at pH 8.4 of non-osmotically protected mitochondria was measured as described in Ref.^56^.

### BN- and SDS PAGE analyses

Blue native-PAGE experiments were carried out as described^57^. Briefly 200 µg of mitochondrial proteins was suspended in 100 µL of extraction buffer (30 mM HEPES pH = 6,8, 150 mM potassium acetate, 12% glycerol, 2 mM 6-aminocaproic acid, 1 mM EGTA, 1.5% digitonin (Sigma)), supplemented with protease inhibitors cocktail tablet (Roche, one tablet per 10 mL of the buffer). After 26 min incubation on ice, the extracts were cleared by centrifugation (21,950×*g*, 4 °C, 30 min), supplemented with 4.5 µL of loading dye (5% Serva Blue G-250, 750 mM 6-aminocaproic acid) and run on NativePAGE™ 3–12% Bis–Tris Gels (Invitrogen). For SDS-PAGE analysis 10 OD of cells was centrifuged, pellet was suspended in 500 µL of 0.2 M NaOH and incubated for 10 min on ice. Then 50 µL of 50% TCA (trichloroacetic acid) was added, vortexed and after 10 min incubation on ice, centrifuged at 21,950×*g* for 10 min at 4 °C. The protein pellet was washed with 1 mL of 1 M Tris-base and suspended in 50 µL of 5% SDS for measurement of protein concentration by method of Lowry^58^. 50 µg of protein were loaded per lane of 10% SDS-PAGE gel^59^. After transfer onto a PVDF or nitrocellulose membrane, ATP synthase complexes were detected using polyclonal antibodies raised against Atp1, Atp2, Atp4, Atp7, Atp17, Atp18, OSCP subunits of yeast ATP synthase at 1:10,000 dilution (a kind gift from Marie-France Giraud, Bordeaux, France)^60^ or against Por1 (gift from prof. Teresa Zoladek, IBB PAS). The membranes were incubated successively with the indicated antibodies—membranes were stripped before incubation with anti-Por1 antibody (Restore™ PLUS Western Blot Stripping Buffer, Thermo Scientific, ref. 46430). Immunoreactivity was studied using secondary antibody conjugated to horseradish peroxidase (DAKO). The blots were stained with Immobilon Western Chemiluminescent HRP Substrate from Millipore (ref. WBKLS0500) used as a substrate. For image acquisition the Uvitec Cambridge Q4 Alliance System (Uvitec Cambridge, UK) was used, images were collected in the serial accumulation option. Images were processed with ImageJ and Adobe Photoshop CC 2019.

### Purification of the ATP synthase holoenzyme from the mitochondria

The protocol described in the Ref.^61^ was applied. 5 mg of mitochondria were centrifuged in 2 mL tubes and suspended in 1 mL of sonication buffer (250 mM saccharose, 50 mM NaH_2_PO_4_, 5 mM 6-aminocaproic acid, 1 mM EDTA, pH 7.5, protease inhibitors cocktail tablet (Roche), 1 µM PMSF) and sonicated 6 times 10 s, with 10 s intervals on ice. After centrifugation of the extract at 5000×*g* for 10 min at 4 °C supernatant was ultracentrifuged at 268,526×*g* during 1 h (Thermo Scientific™ Sorvall™ WX ultracentrifuge, TFT80.2 rotor). The pellet was washed twice with the sonication buffer without EDTA (without suspending it) and then suspended with the use of the potter in 500 µL of MP extraction buffer (150 mM potassium acetate, 10% glycerol, 2 mM 6-aminocaproic acid, 30 mM HEPES, pH 7.4, 1% *N*-dodecyl-β-maltoside, 2 mM PMSF and protease inhibitors cocktail tablet) by 20 min incubation on ice. The membranes were centrifuged 30 min at 21,950×*g* at 4 °C and the extract was incubated with 200 µL of the Ni-NTA agarose washed previously by Binding buffer (50 mM NaCl, 10% glycerol, 10 mM imidazole, 20 mM NaH_2_PO_4_, pH = 7.9, 0,1% *n*-dodecyl-β-maltoside, 2 mM PMSF, protease inhibitors cocktail tablet) for the night. Next day the beads were washed twice with the Binding buffer, then suspended in 400 µL of Binding buffer, dosed and after addition of 100 µL of 5 × Laemmli sample buffer, boiled 5 min. The 50 µg of the extract and 2 µg of the bead’s eluate were loaded on the 15% gel.

### In silico analysis of subunit* 8* substitutions in the ATP synthase structures

Multiple sequences of ATP synthase *8*-subunits of various origins were aligned and drawn using Clustal Omega^62^ and Espript 3.0^63^. Molecular views of ATP synthase monomer and the F_O_ subunits *8*, *a*,* j* and *c*-ring were obtained from the monomer of bovine ATP synthase (pdb_id: 6zbb^28^). The pdb records: 6cp5 and 6zbb were used as the starting points for the in silico analysis of yeast and mammalian ATP synthase F_O_ domains, respectively^35^ using Yasara Structure package (http://www.yasara.org/). The side-chain conformations were iteratively optimized for both structures with ten cycles of repairPDB procedure implemented in FoldX5 (http://foldxsuite.crg.eu/). The original sequence of subunit *8* of bovine ATP synthase was then replaced with a *Homo sapiens* one (A0A075X6N5_HUMAN), and the side-chain packing in the resulting structure was again re-optimized with FoldX5. The effect of single-residue replacement was analyzed in silico using FoldX5^64^. The thermodynamic effect of residue replacement at a given position (ΔΔG, expressed in kcal/mol) was then assessed for the whole F_O_ domain with FoldX5 using PositionScan, and further decomposed to the contribution of particular intra- and inter-domain interaction using AnalyseComplex. The stability (ΔG) of a protein is defined by the free energy. The lower it is, the more stable a protein is. The ΔΔG is a difference in free energy between a wild-type and substituted variant. A substitution that brings energy (ΔΔG > 0 kcal/mol) will destabilize the protein structure, while a substitution that remove energy (ΔΔG < 0 kcal/mol) will stabilize the protein structure. A common threshold ΔΔG > 1 kcal/mol is considered as a significant effect. The structure figures were drawn using PyMOL™ 2.3.2 (https://pymol.org/2/) or Yasara Strucure package (http://www.yasara.org/)^65^.

### Statistical analysis

Three biological and three technical replicates were performed for all experiments. The unpaired two-tailed *t* test was used for all data sets. Significance and confidence level was set at 0.05.

### Statement of ethics

The permission number for work with genetically modified microorganisms (GMM I) for RK is 01.2-28/201.

## Supplementary Information

Supplementary Information.

Supplementary Figures.

## Data Availability

Supplementary data and the materials (yeast strains, sequence of the *ATP8* locus amplified by PCR using the mtDNA from the model strains) are available upon request from the corresponding author.
