# Supplementary material for: Analysis of MT-ATP8 gene variants reported in patients by modeling in silico and in yeast model organism
Source: Sci Rep. 2023 Jun 20;13:9972. doi: 10.1038/s41598-023-36637-9 (PMC10282124; doi:10.1038/s41598-023-36637-9)
Supplement: Supplementary file 1 — Supplementary Information. [file 41598_2023_36637_MOESM1_ESM.docx]

**Analysis of *MT-ATP8* gene variants reported in patients by modeling *in silico* and in yeast model organism**

Chiranjit Panja, Katarzyna Niedzwiecka, Emilia Baranowska, Jaroslaw Poznanski, Roza Kucharczyk*

Institute of Biochemistry and Biophysics, Polish Academy of Sciences, Warsaw, Poland

*Corresponding author: roza@ibb.waw.pl

Keywords: mitochondrial diseases, neuropathy, cardiomyopathy, mtDNA, subunit *8*, ATP synthase

Supplementary material

**Table S1** Influence of the *8*L_13_T substitution on yeast mitochondrial respiration, ATP synthesis and hydrolysis activities in strain bearing wild type subunit *a*/Atp6.

| 28 °C | | | | | | | | | |
| --- | --- | --- | --- | --- | --- | --- | --- | --- | --- |
| Strain | Respiration rates  nmol O.min^-1^.mg^-1^ | | | | ATP synthesis rate  nmol ATP/min^-1^/mg^-1^ | | P/O | ATPase activity  µmol Pi.min^-1^.mg^-1^ | |
|  | NADH  State 4 | NADH +ADP  State 3 | NADH +CCCP | Asc/TMPD  + CCCP | - oligo | + oligo |  | - oligo | +oligo |
| *a*WT *8*WT | 683±156 | 1277±160 | 2138±102 | 4943±74 | 1507±82 | 233±86 | 1.18± 0.06 | 2.934±0.213 | 0.384±0,082 |
| *a*WT *8*L_13_T | 706± 63 | 1365 ±136 | 1865 ± 89 | 5277 ±462 | 1540±5 | 227±20 | 1.13±0.11 | 2.602±0.064 | 0.236± 0.068 |
| 36 °C | | | | | | | | | |
| *a*WT *8*WT | 302±9 | 632±67 | 1101±101 | 3640±107 | 906±112 | 239±6 | 1,43± 0,02 | 1.879±0.041 | 0.668±0,143 |
| *a*WT *8*L_13_T | 300±6 | 668±12 | 1168±98 | 3683±19 | 804±55 | 302±44 | 1,2±0,06 | 1.795±0.014 | 0.601± 0.064 |

**Table S2** Influence of the *8*L_13_T substitution on yeast mitochondrial respiration, ATP synthesis and hydrolysis activities in strain bearing HisHA tag fused to *a*/Atp6.

| 28 °C | | | | | | | | | |
| --- | --- | --- | --- | --- | --- | --- | --- | --- | --- |
| Strain | Respiration rates  nmol O.min^-1^.mg^-1^ | | | | ATP synthesis rate  nmol ATP/min^-1^/mg^-1^ | | P/O | ATPase activity  µmol Pi.min^-1^.mg^-1^ | |
|  | NADH  State 4 | NADH +ADP  State 3 | NADH +CCCP | Asc/TMPD  + CCCP | - oligo | + oligo |  | - oligo | +oligo |
| *a*HisHA *8*WT | 732±23 | 1501±62 | 2206±94 | 5352±193 | 1646±36 | 54±6 | 1.1±0.02 | 3.803 ± 0.115 | 0.613±0,104 |
| *a*HisWT *8*L_13_T | 459±22* | 871 ±35* | 1343 ±31* | 3137 ±376* | 1076±133* | 53±2 | 1.24±0.1 | 3.525 ± 0.260 | 0.707± 0.71 |
| 36 °C | | | | | | | | | |
| *a*HisHA *8*WT | 262±15 | 552±45 | 920±99 | 1970±97 | 646±85 | 126±35 | 1,17±0,06 | 3.017 ± 0.038 | 0.358±0,30 |
| *a*HisWT *8*L_13_T | 144±7* | 299±14* | 478±5* | 1503±111* | 404±21* | 27,2±5 | 1,35±0,01 | 2.563* ± 0.189 | 0.351± 0.023 |

Mitochondria were isolated from wild type and mutants strains grown for 5-6 generations in YPGalA at 28°C or 36°C. All cultures grown at 28°C contained 2-5 % of ρ-/ρ° cells, while those grown at 36°C had 30-42% of ρ-/ρ° cells. Reaction mixes for assays contained 0.075 mg/ml of protein, 4 mM NADH, 150 (for respiration assays) or 750 (for ATP synthesis) μM ADP, 12.5 mM ascorbate (Asc), 1.4 mM N,N,N,N,-tetramethyl-p-phenylenediamine (TMPD), 4 μM CCCP, 3 μg/ml oligomycin (oligo). For the ATPase assays, mitochondria kept at –80°C were thawed and the reactions were performed in the absence of osmotic protection and at pH 8.4. The values reported are averages of triplicate assays ± standard deviation. * indicates statistical significance set at p<0.05.

**Figure S1.** Growth curves in liquid media. Cultures were inoculated from liquid rich glucose grown cells. The experiments were performed in triplicate and the representative plates and curves are shown. Statistical significance is indicated: *p<0.05.
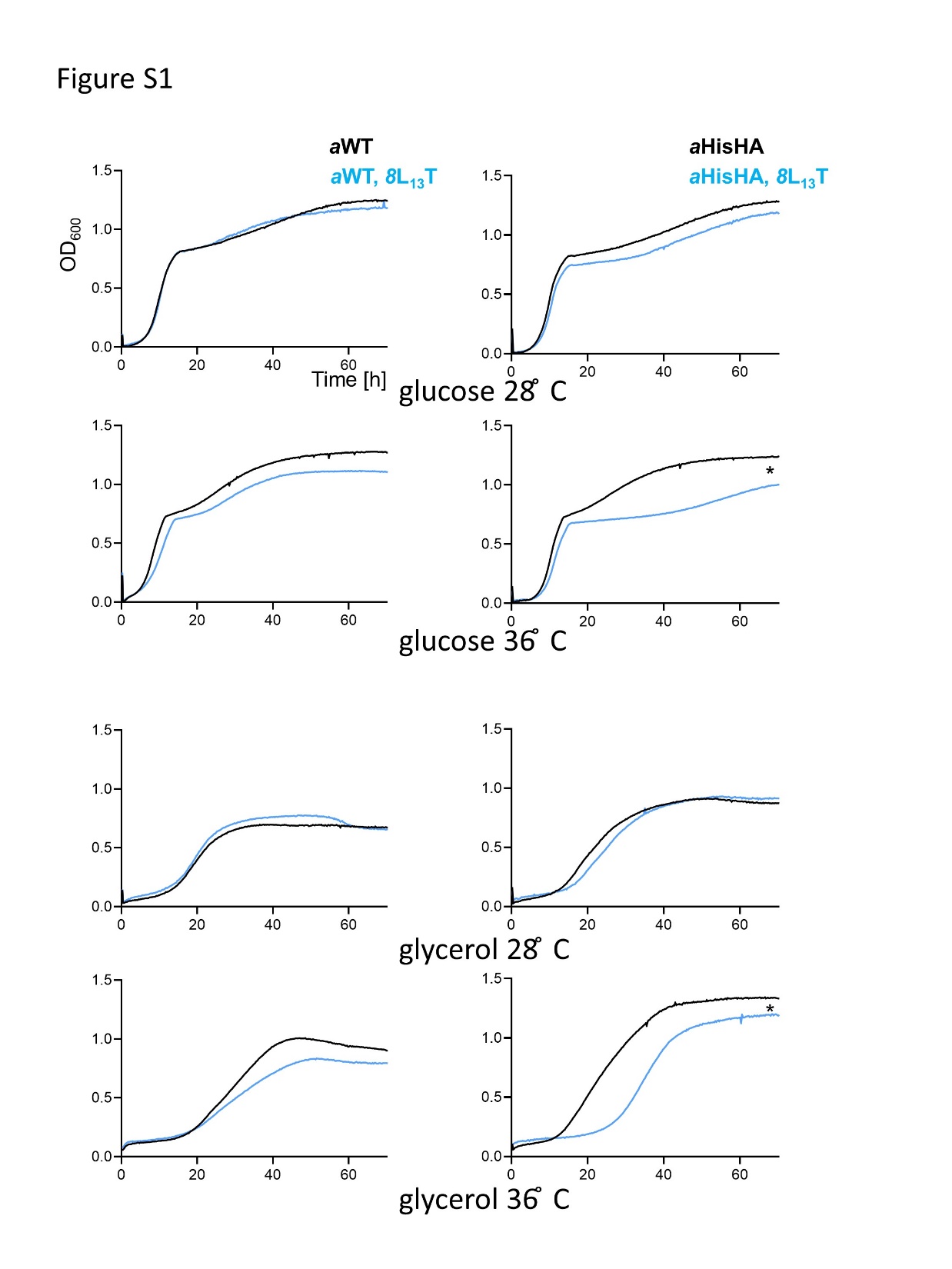


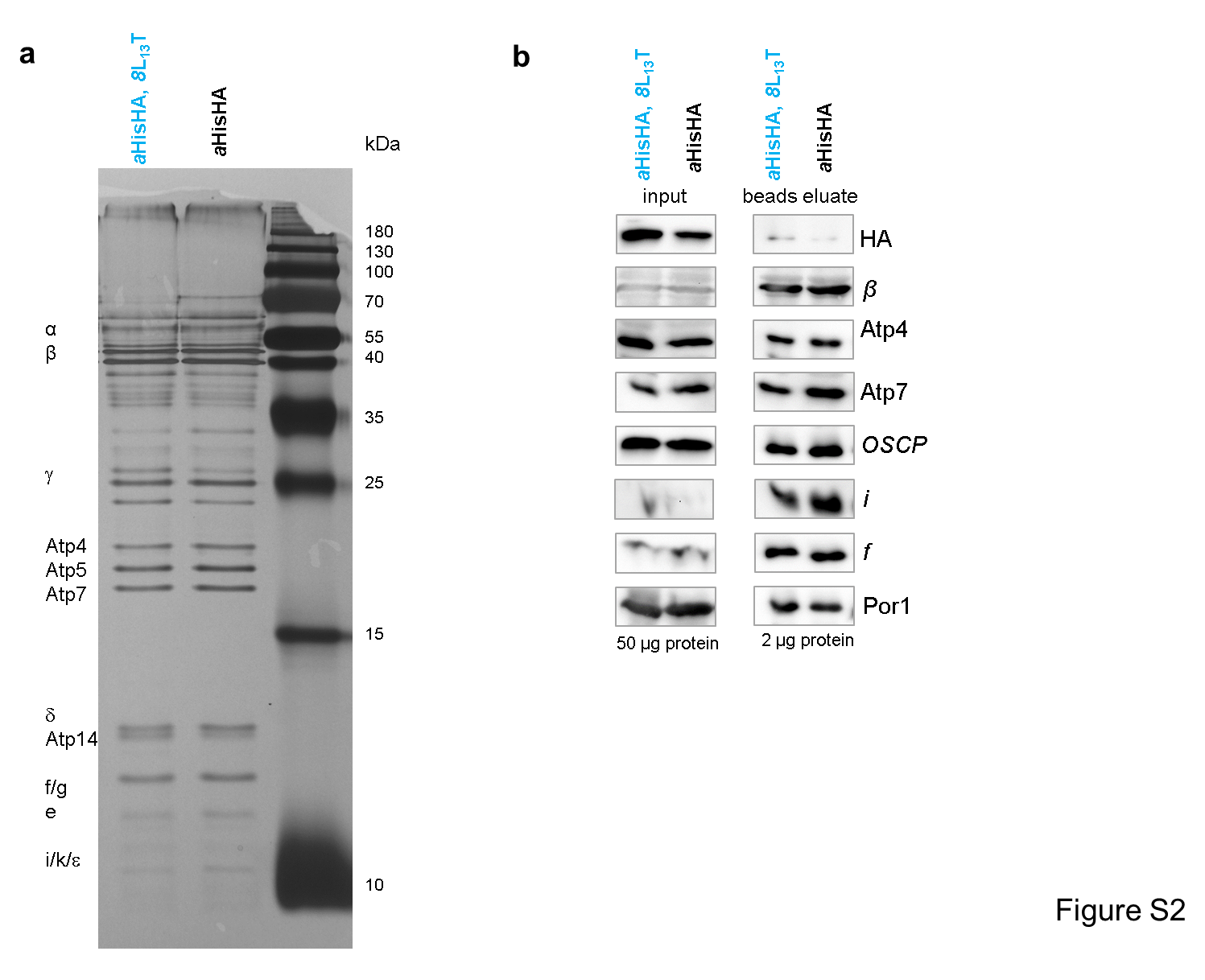


**Figure S2.** Purification of the ATP synthase complexes from the mitochondrial membrane of *8*L_13_T *a*HisHA cells. **a**: After extraction the ATP synthase complexes were pulled-down by HisHA-tagged subunit *a*, the eluates were separated in 15 % SDS-PAGE and the bands were visualized by silver staining. The dominating proteins identified in bands by mass spectrometry are indicated on the left. The representative silver stained gel is shown. **b**: Amount of indicated subunits in purified ATP synthase complex. The proteins bound to the Ni-NTA beads were solubilized in 5x Laemmli sample buffer by boiling. The 50 µg of the extract and 2 µg of the beads eluate was loaded on the 15 % SDS-PAGE gel. After transfer membranes were incubated with the antibodies against indicated subunits or porin as a loading control. The experiment was performed many times and the representative gels are shown. The original blots are presented in SupplementaryRowImages pages 6-17.
