# Supplementary material for: Analysis of MT-ATP8 gene variants reported in patients by modeling in silico and in yeast model organism
Source: Sci Rep. 2023 Jun 20;13:9972. doi: 10.1038/s41598-023-36637-9 (PMC10282124; doi:10.1038/s41598-023-36637-9)
Supplement: Supplementary file 2 — Supplementary Figures. [file 41598_2023_36637_MOESM2_ESM.pdf]

Supplementary Data – Row Images of Figures 6 and S2

Analysis of *MT-ATP8* gene variants reported in patients by modeling *in silico* and in yeast model organism

Chiranjit Panja, Katarzyna Niedzwiecka, Emilia Baranowska, Jaroslaw Poznanski, Roza Kucharczyk*
Institute of Biochemistry and Biophysics, Polish Academy of Sciences, Warsaw, Poland
*Corresponding author: roza@ibb.waw.pl
Keywords: mitochondrial diseases, neuropathy, cardiomyopathy, mtDNA, subunit *8*, ATP synthase

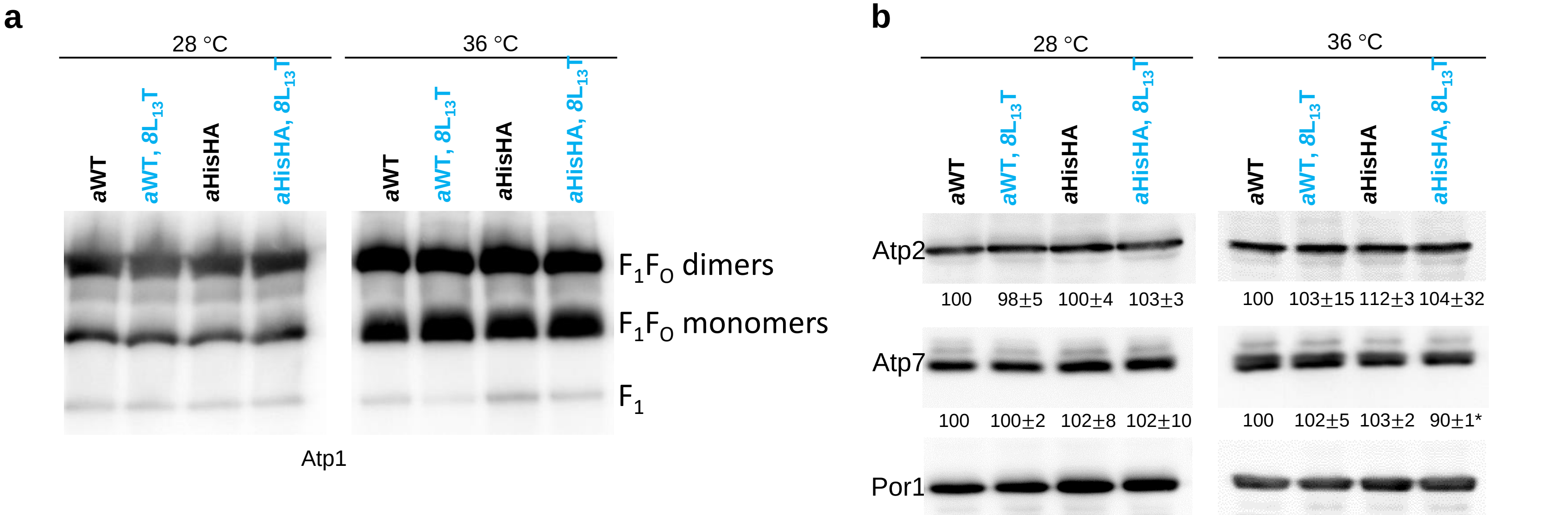
a
28 °C
36 °C
aWT
aWT, 8L13T
aHisHA
aHisHA, 8L13T
F1FO dimers
F1FO monomers
F1
Atp1
b
28 °C
36 °C
aWT
aWT, 8L13T
aHisHA
aHisHA, 8L13T
Atp2
100
98±5
100±4
103±3
100
103±15
112±3
104±32
Atp7
100
100±2
102±8
102±10
100
102±5
103±2
90±1*
Por1

Figure 6

Whole membranes used to prepare Figure 6 panel A

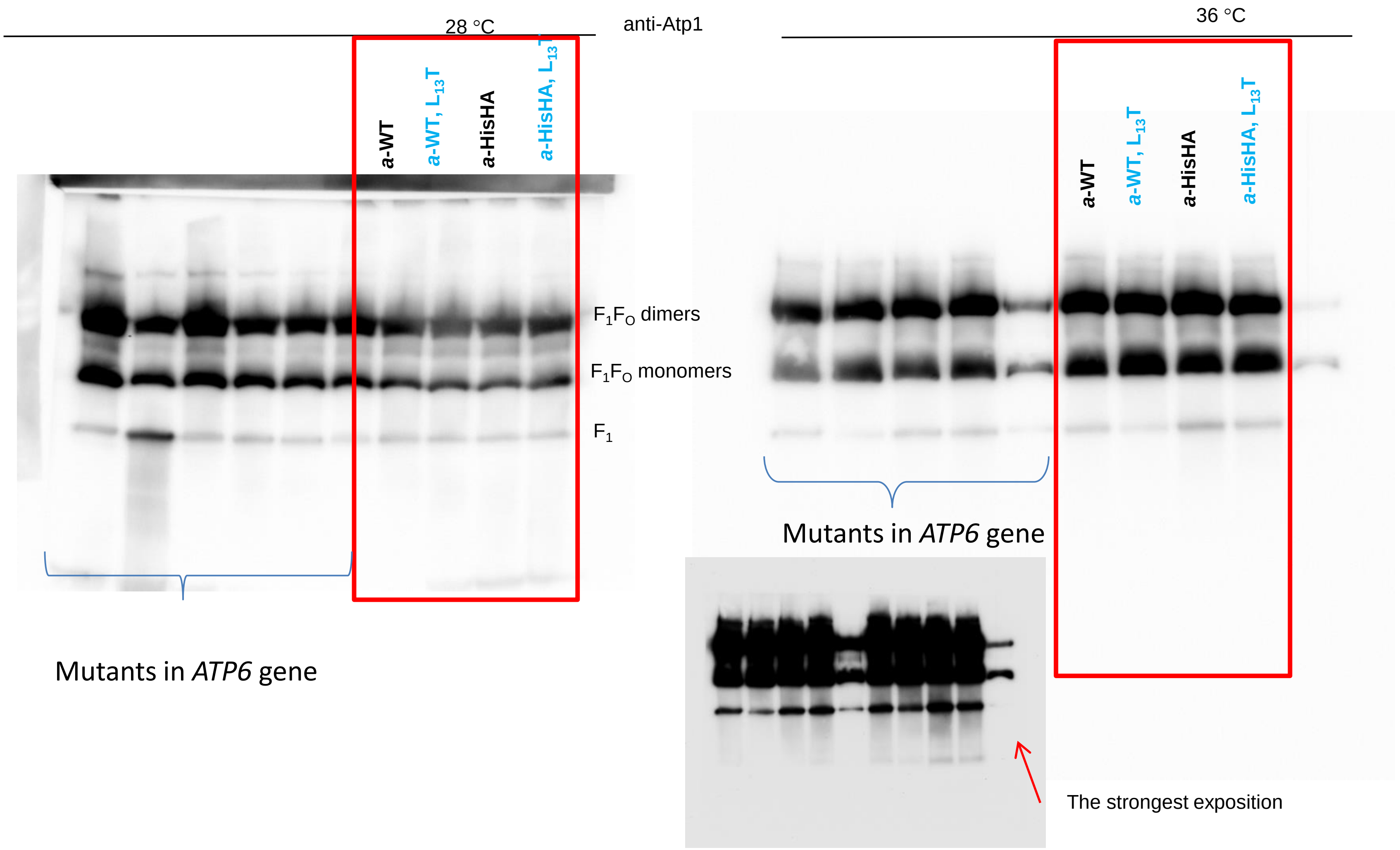

Whole membrane (one) used to prepare Figure 6 panel B – 28°C

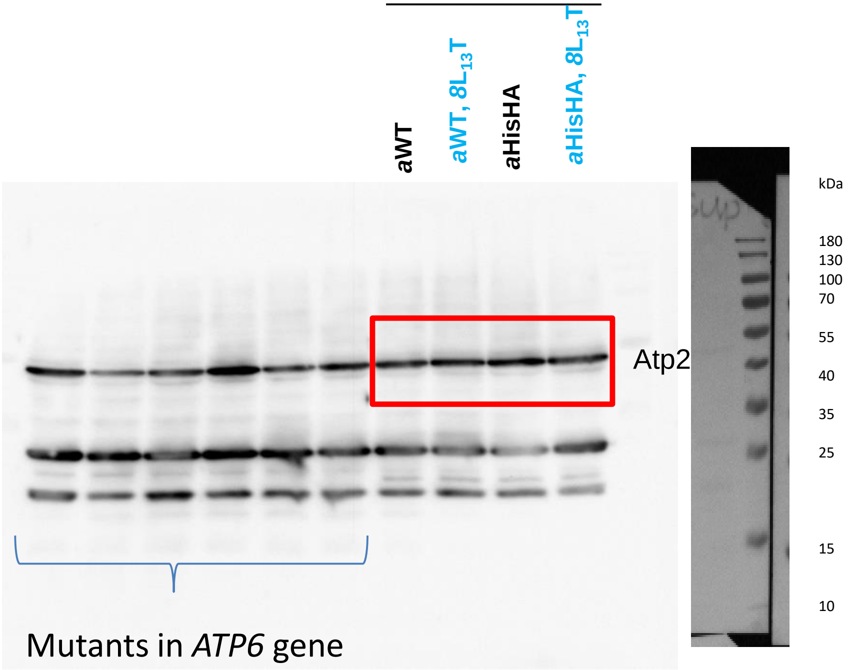

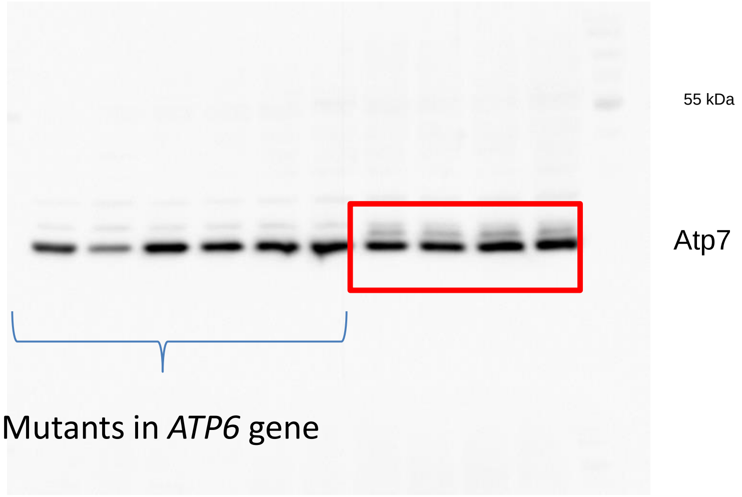

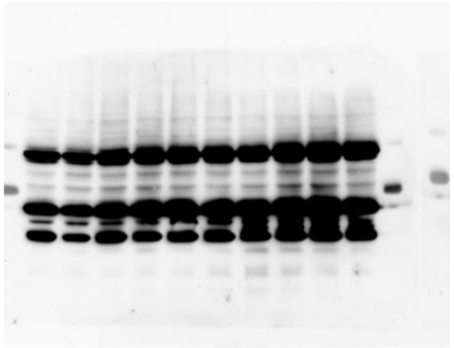

The strongest exposition
of anti-Por1, when gel borders are visible

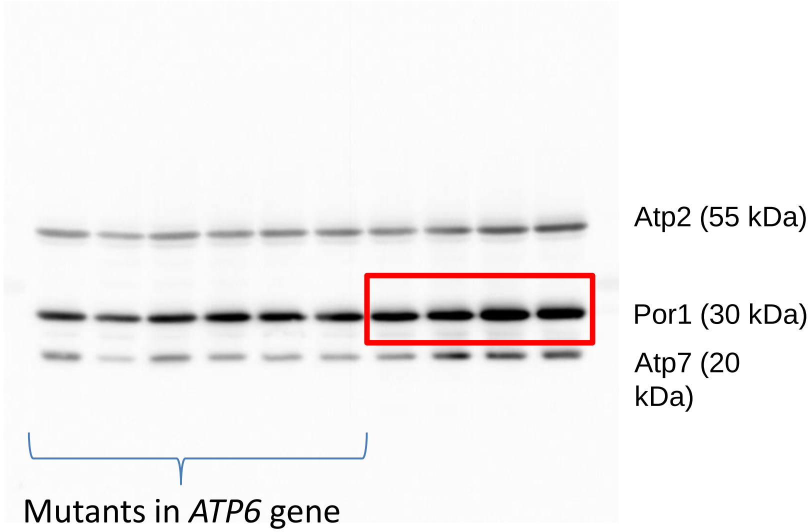

Whole membrane (one) used to prepare Figure 6 panel B – 36°C

36 °C

aWT
aWT, $8L_{13}T$
aHisHA
aHisHA, $8L_{13}T$

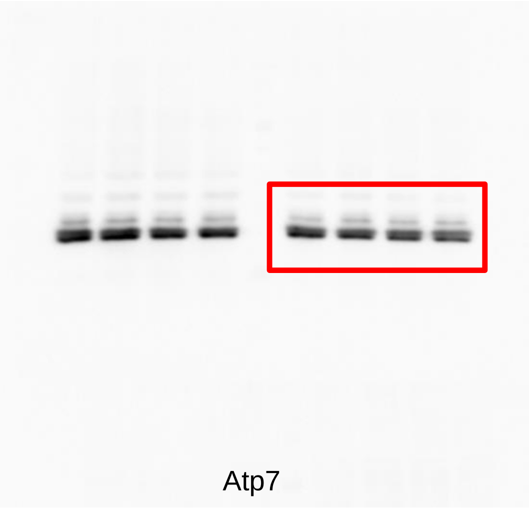

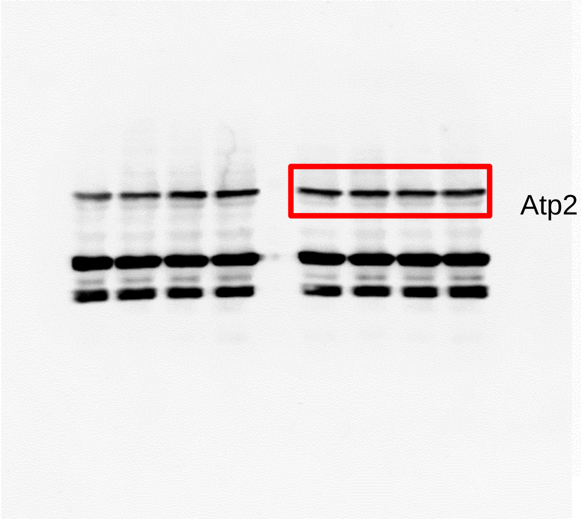

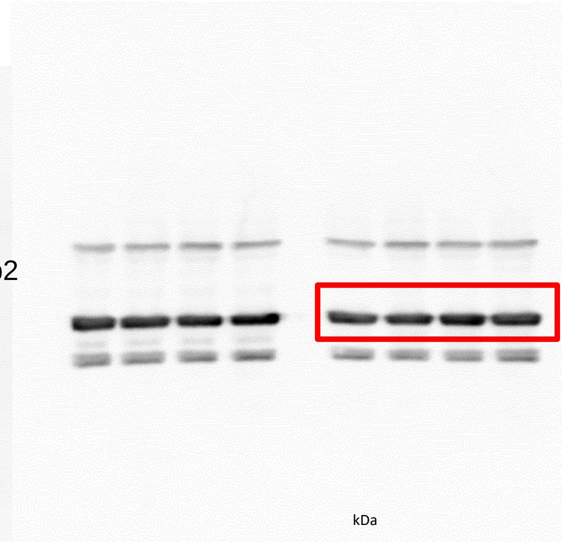

Atp2 (55 kDa)

Por1 (30 kDa)

Atp7 (20 kDa)

kDa

180
130
100
70
55
40
35
25
15
10

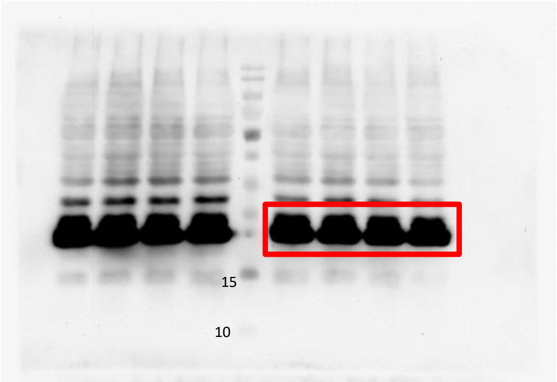

The strongest exposition
of anti-Atp7, when gel borders are visible

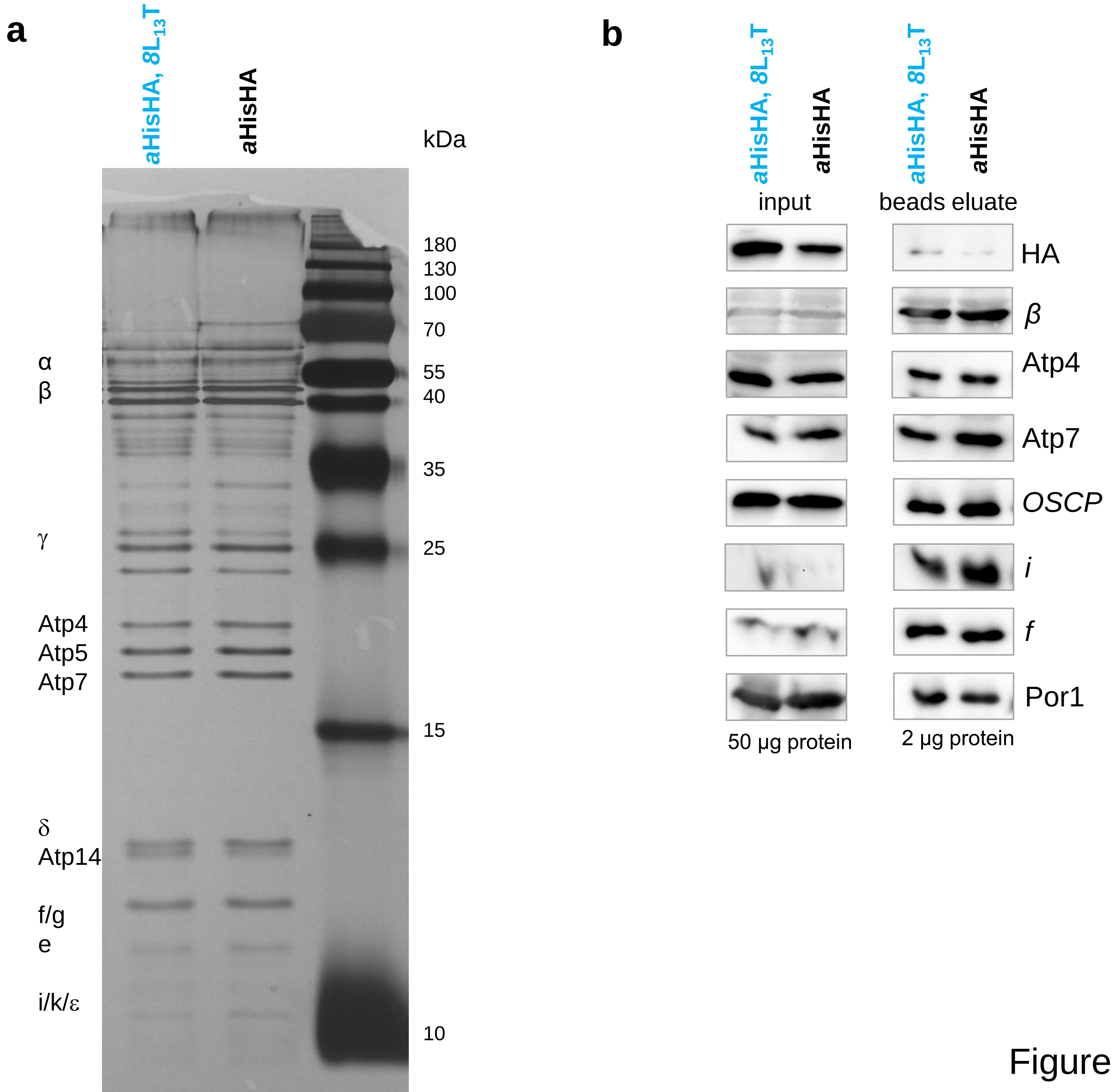
a
aHisHA, 8L13T
aHisHA
kDa
180
130
100
70
55
40
35
25
15
10
α
β
γ
Atp4
Atp5
Atp7
δ
Atp14
f/g
e
i/k/ε
b
aHisHA, 8L13T
aHisHA
aHisHA, 8L13T
aHisHA
input
beads eluate
HA
β
Atp4
Atp7
OSCP
i
f
Por1
50 µg protein
2 µg protein

Figure S2

The whole gel of figure S2a

a

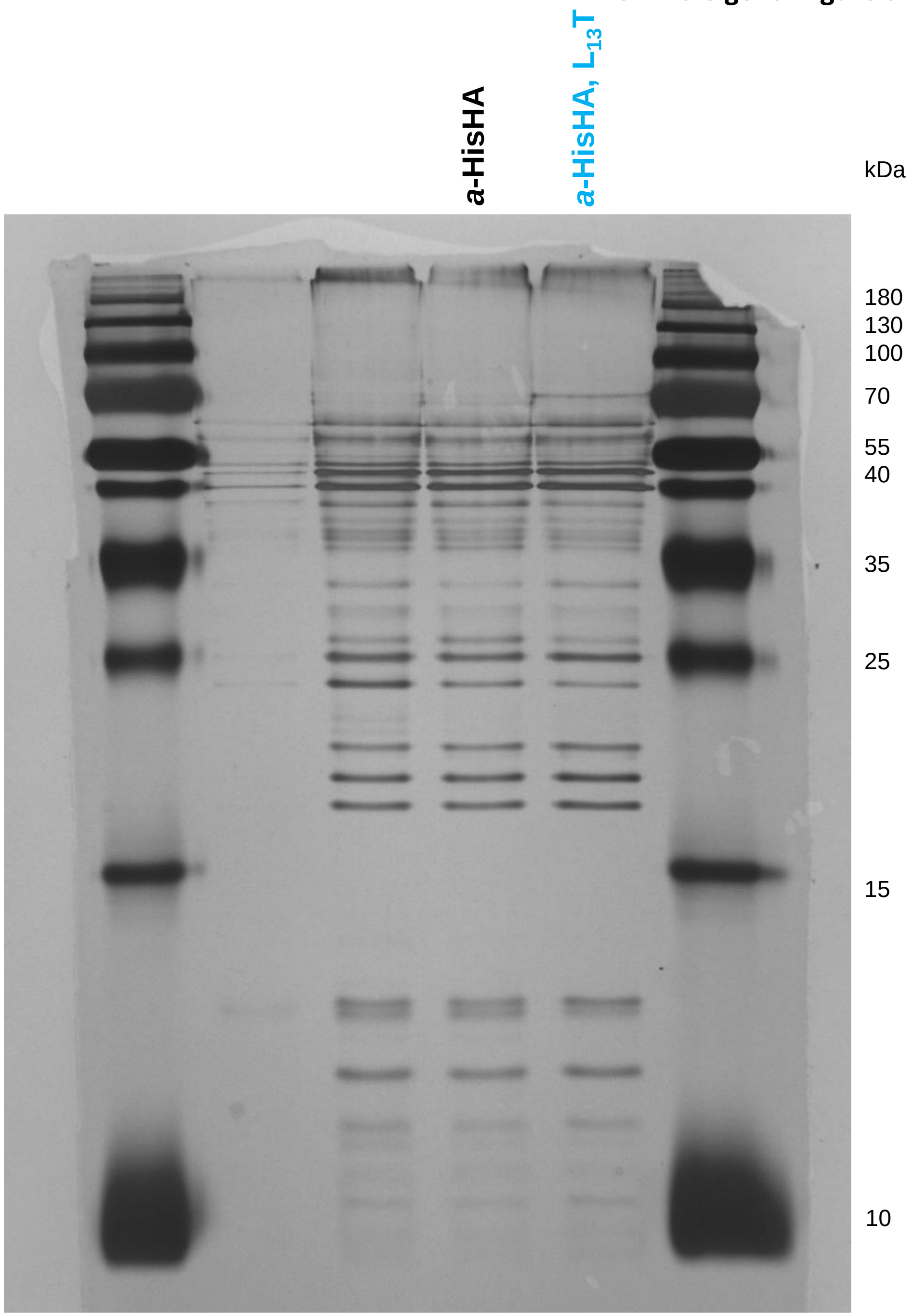

The whole membranes of figure S2b

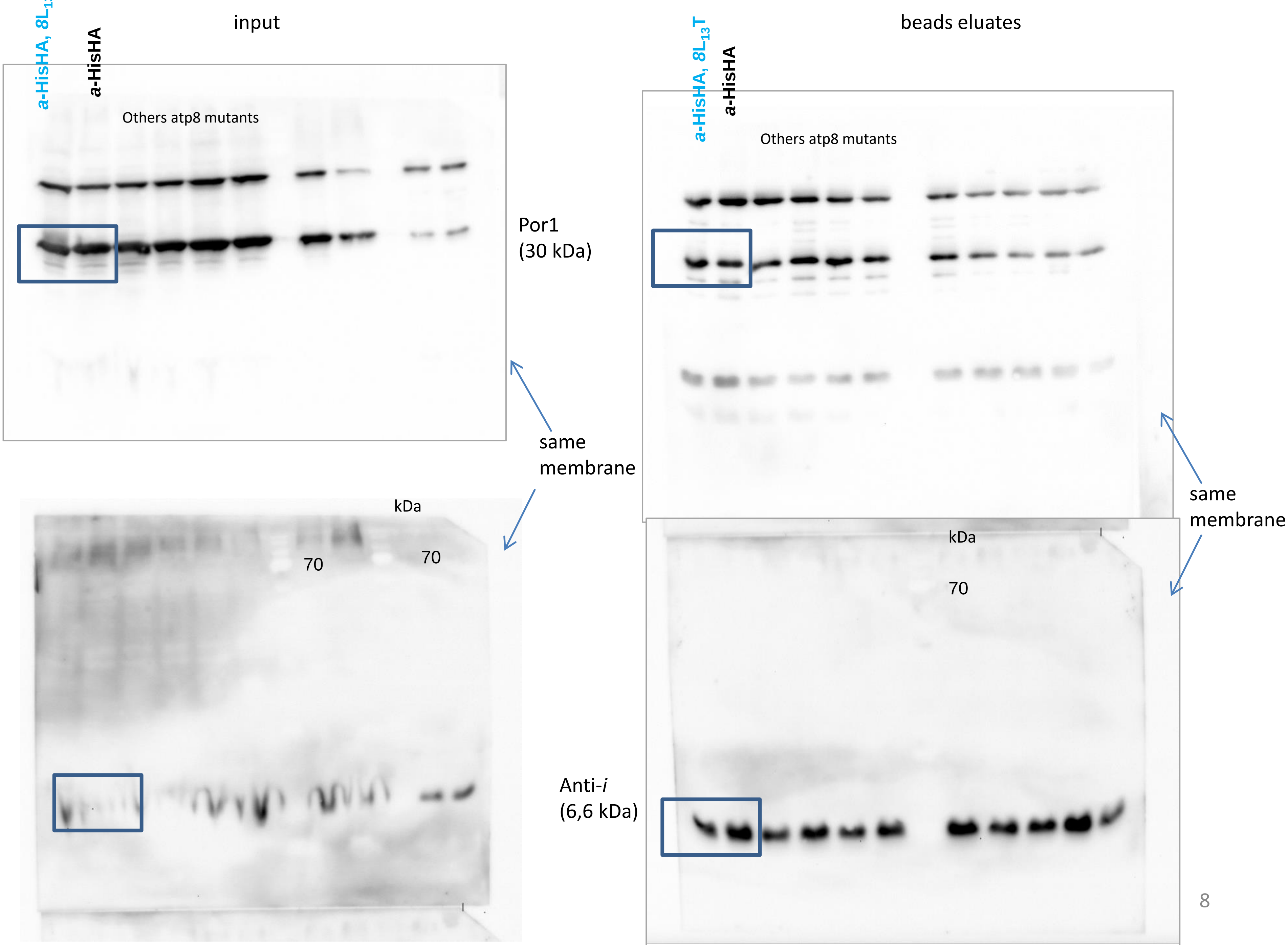

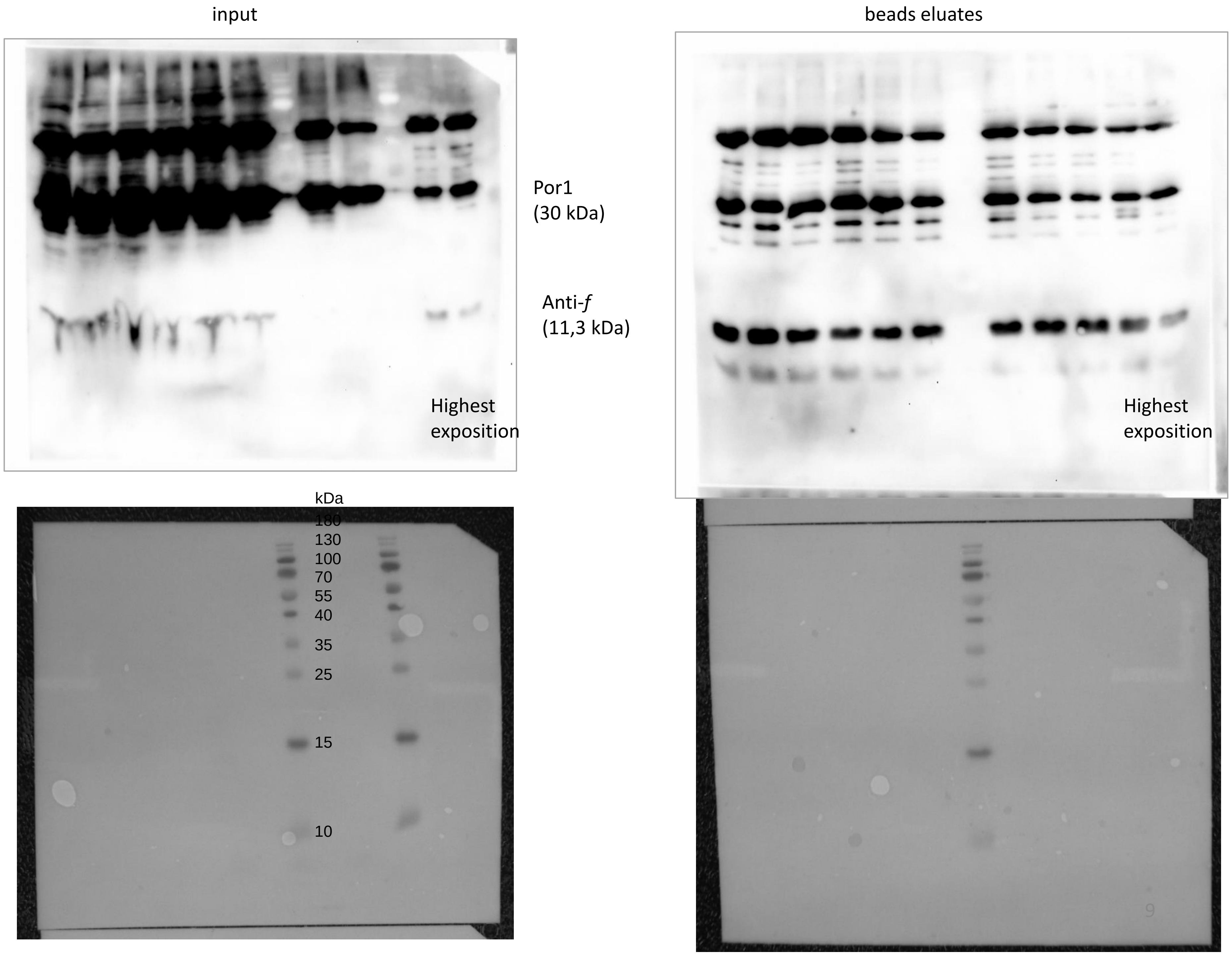
input
beads eluates
Por1
(30 kDa)
Anti-*f*
(11,3 kDa)
Highest
exposition
Highest
exposition
kDa
180
130
100
70
55
40
35
25
15
10

input

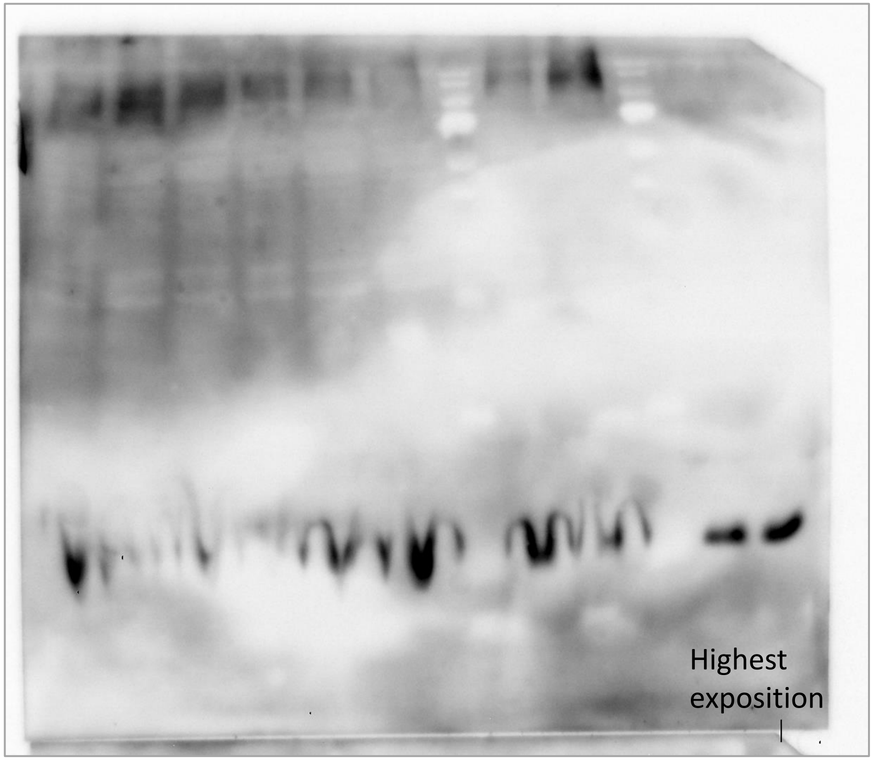

Anti-*i*
(6,6 kDa)

beads eluates

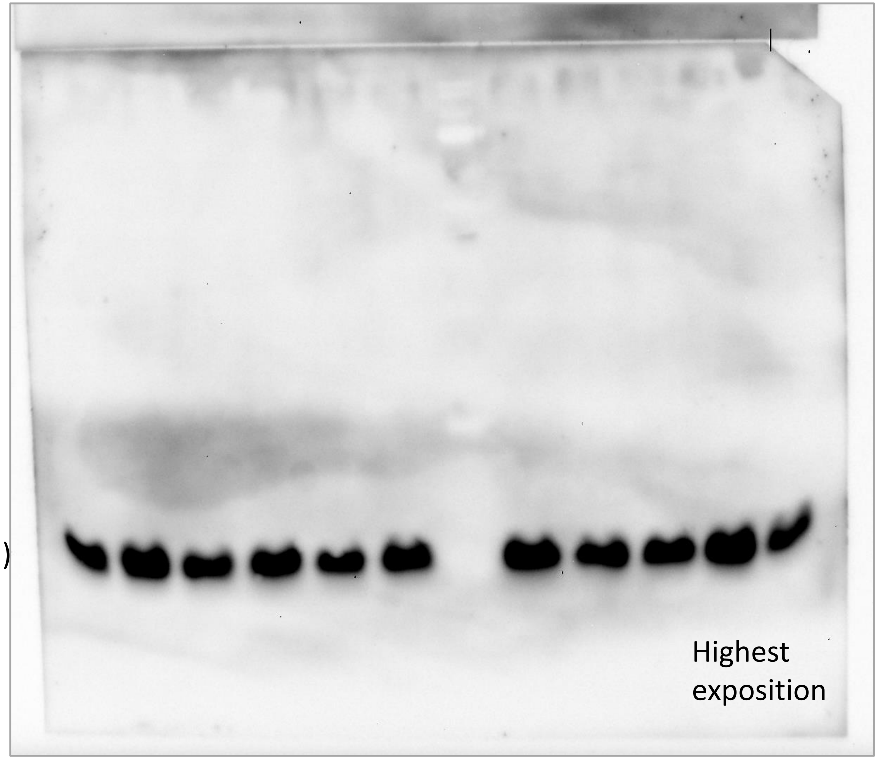

input

beads eluates

a-HisHA, $8L_{13}T$

a-HisHA

Another membrane,
Samples from other mutants

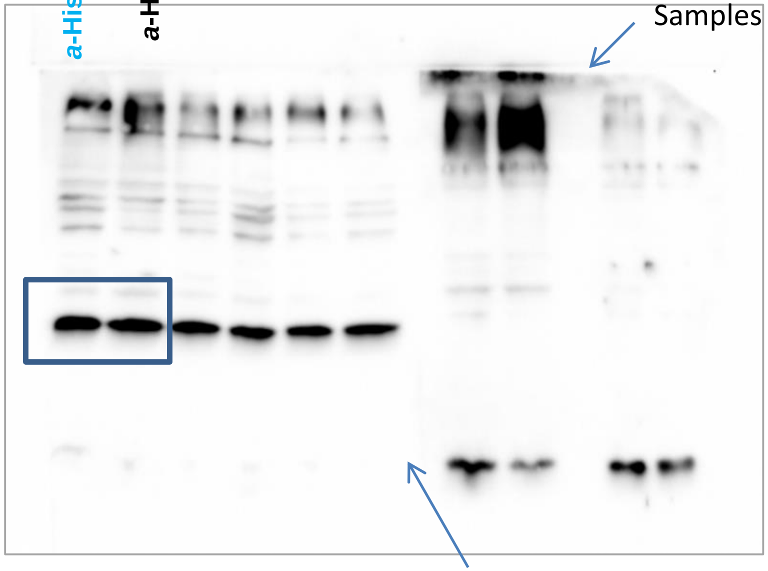

Anti-*OSCP*
(23 kDa)

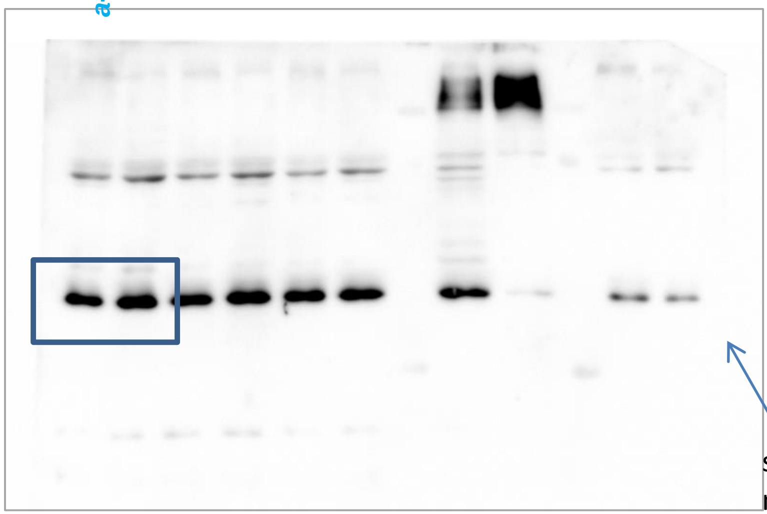

same
membrane

same
membrane

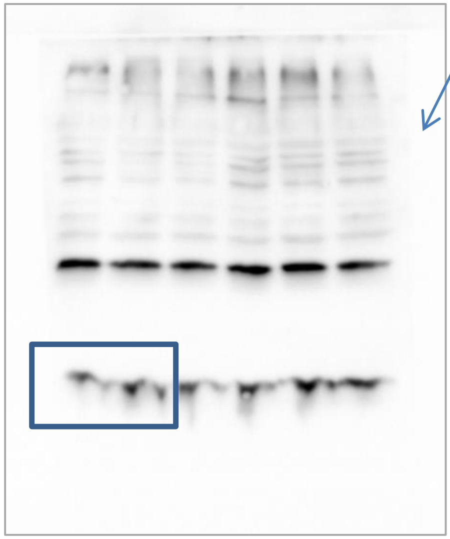

*Unspecific band(s) recognised by anti-*f* antibody

Anti-*f*
(11,3 kDa)

Anti-Atp7

Anti-*f*

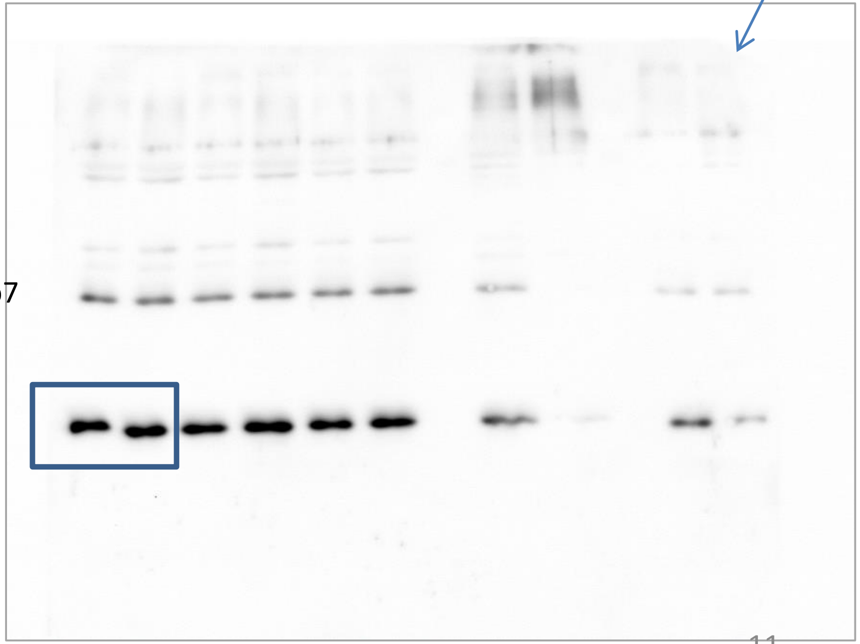

input

beads eluates

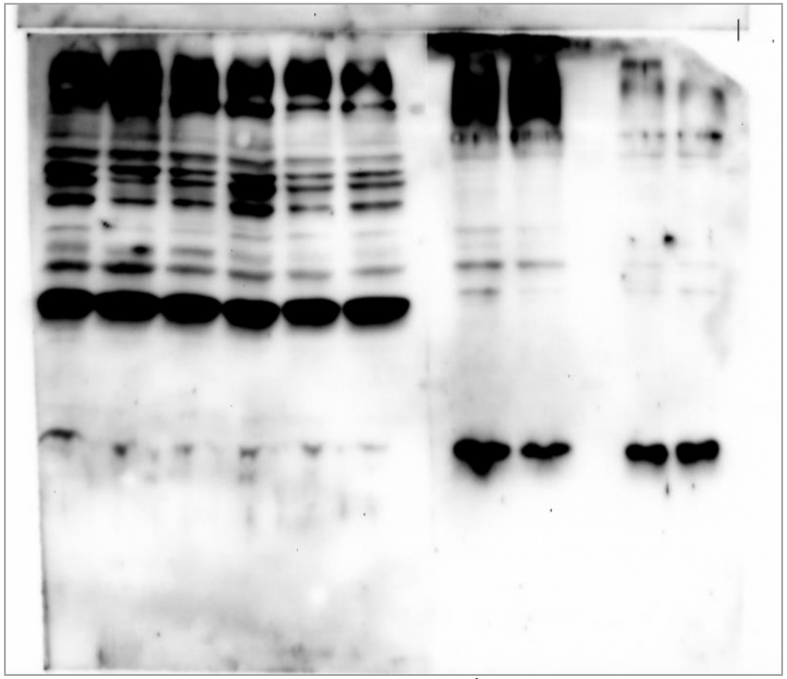

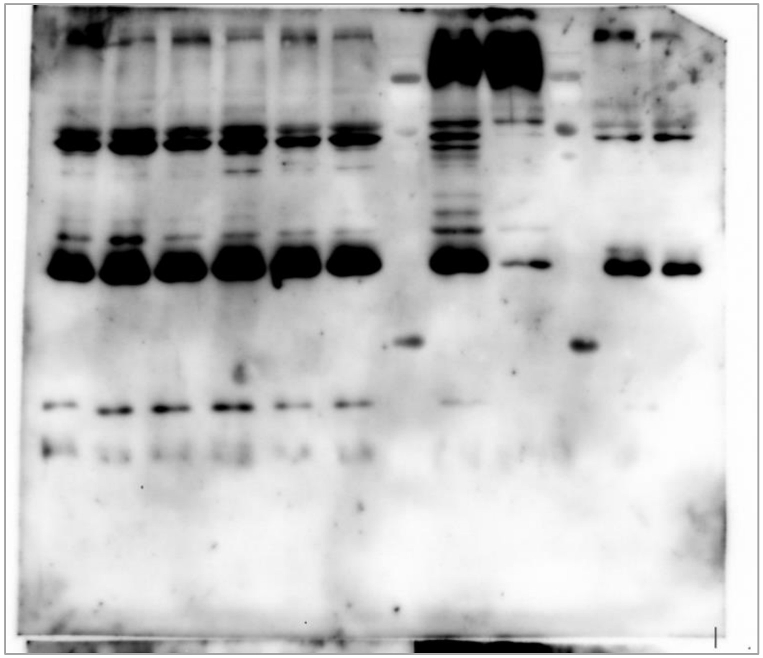

Anti-*OSCP*
(23 kDa)

kDa

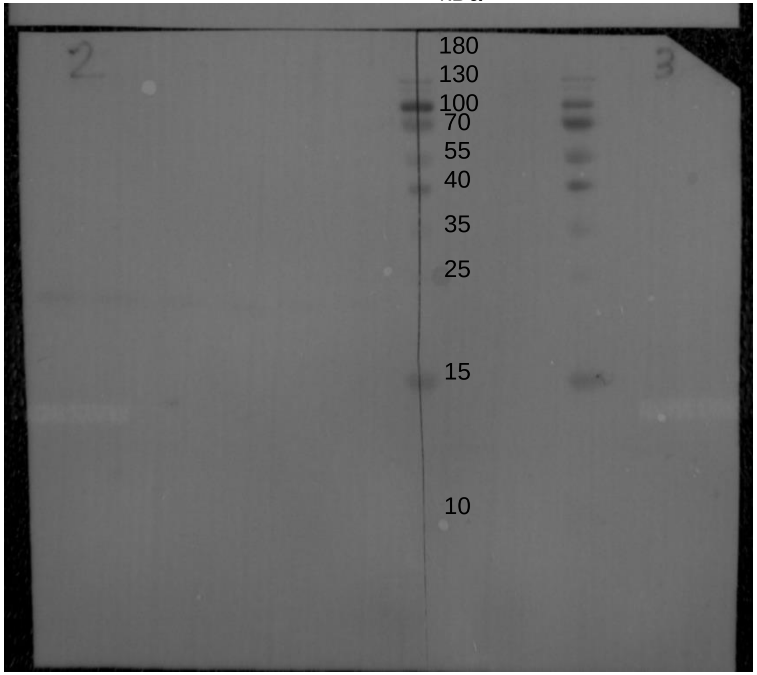

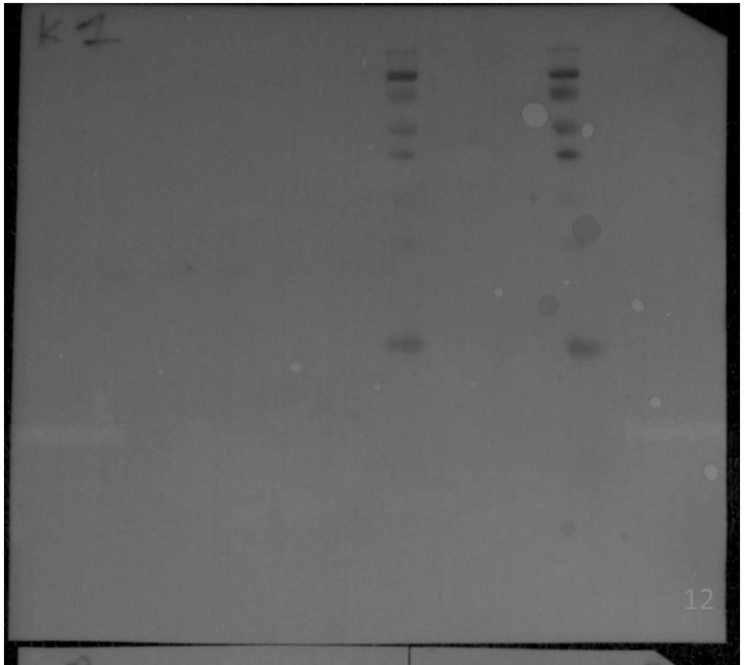

input

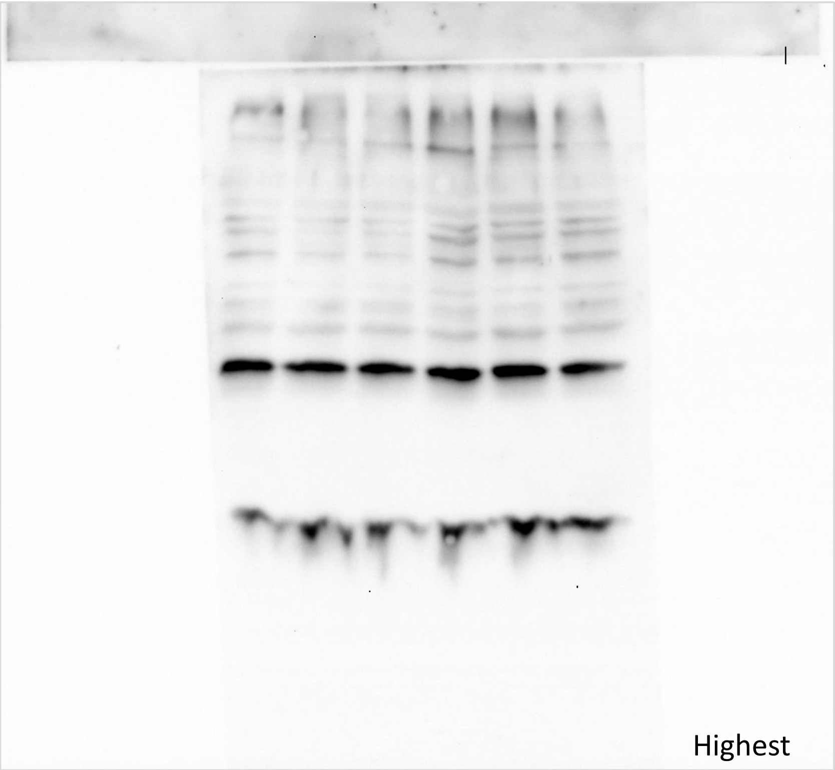

Anti-*f*
(11,3 kDa)

beads eluates

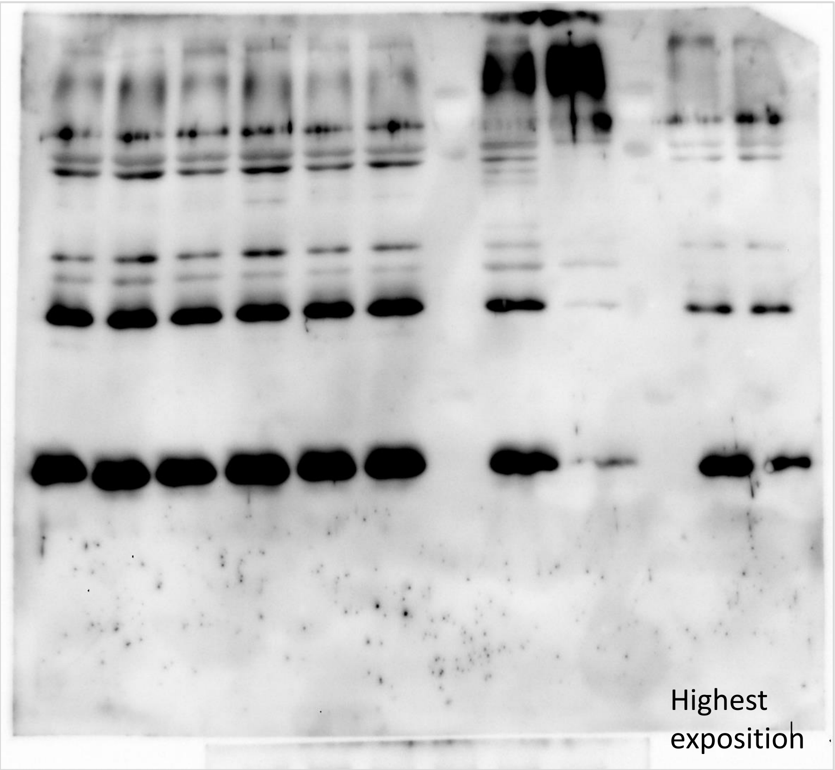

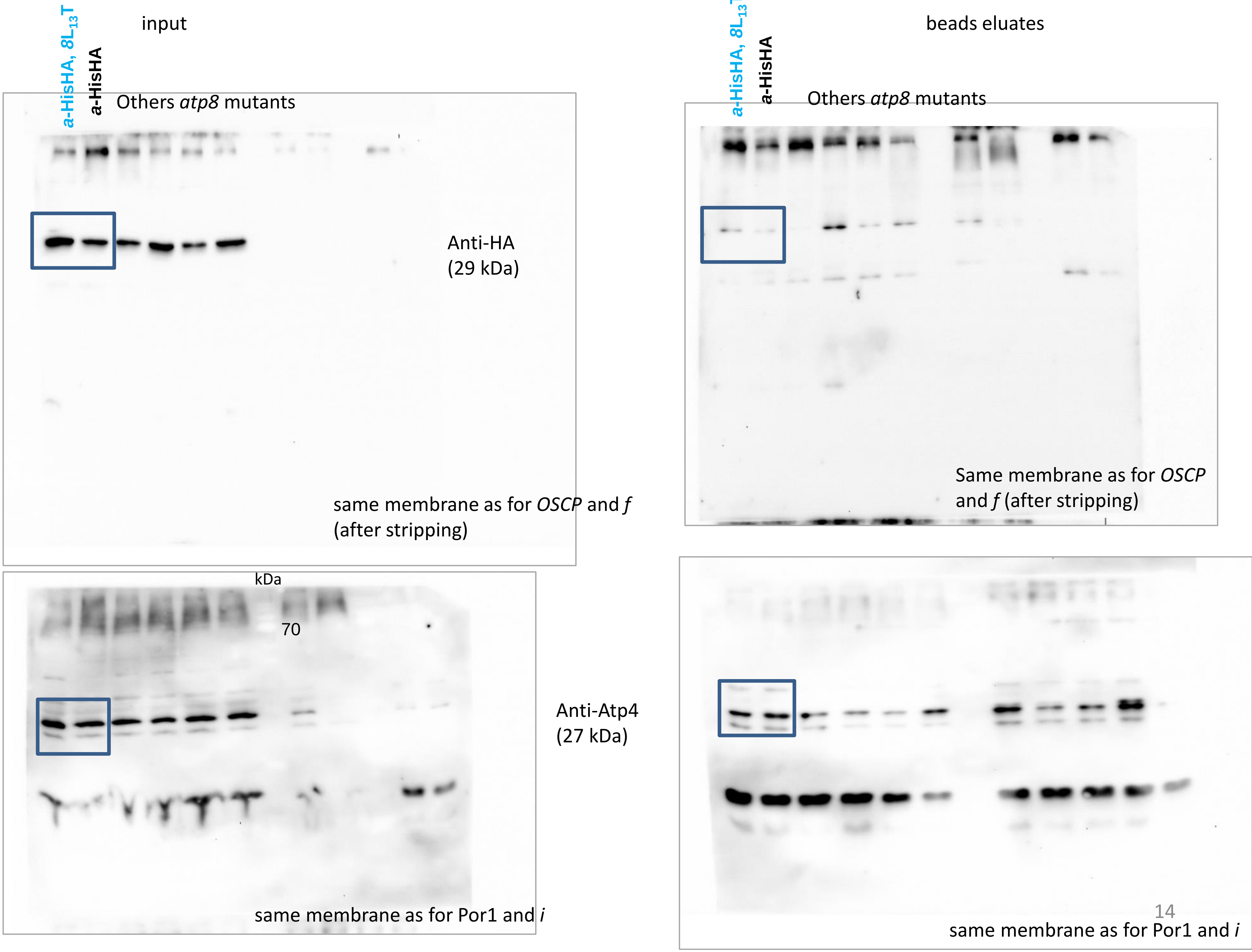
input
a-HisHA, $8L_{13}T$
a-HisHA
Others *atp8* mutants
Anti-HA
(29 kDa)
same membrane as for *OSCP* and *f*
(after stripping)
kDa
70
Anti-Atp4
(27 kDa)
same membrane as for Por1 and *i*
beads eluates
a-HisHA, $8L_{13}T$
a-HisHA
Others *atp8* mutants
Same membrane as for *OSCP* and *f* (after stripping)
same membrane as for Por1 and *i*

input

beads eluates

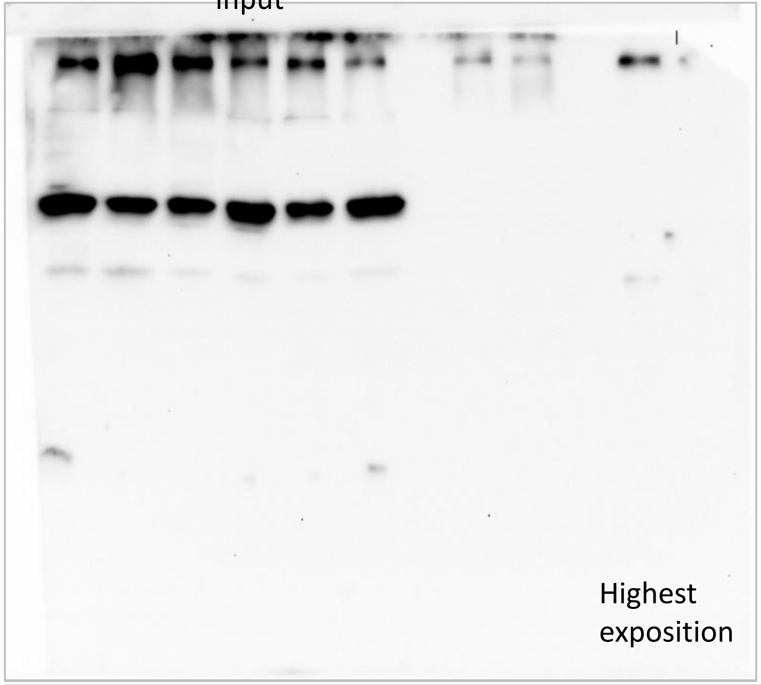

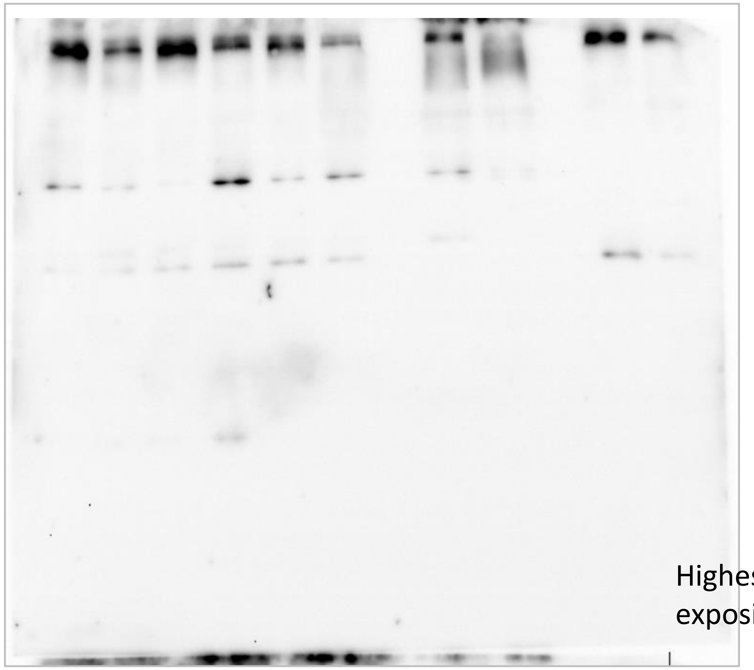

Anti-HA
(29 kDa)

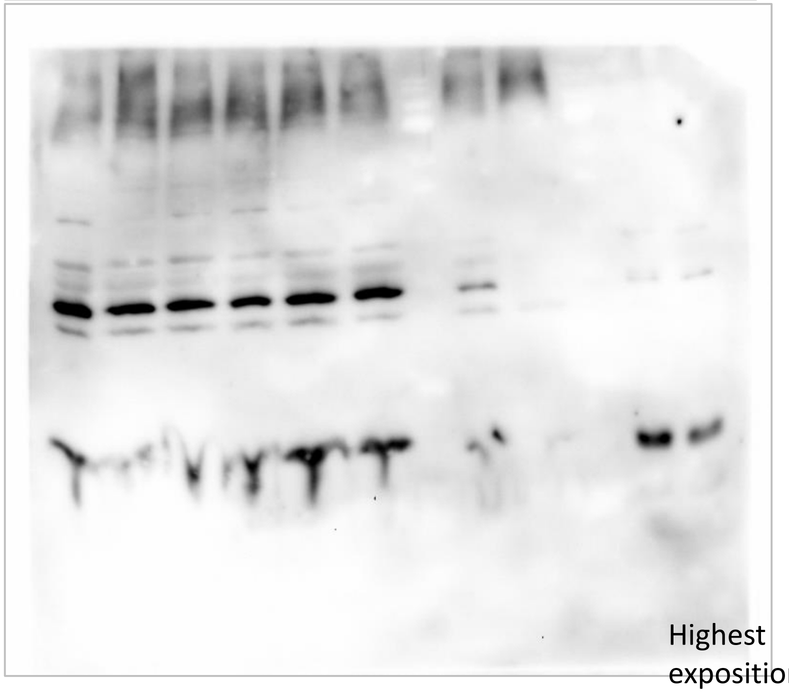

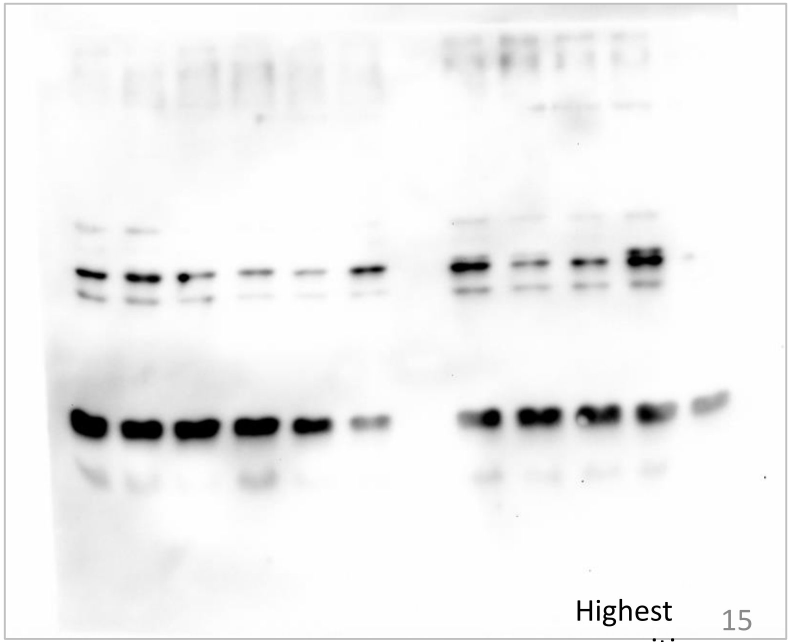

Anti-Atp4
(27 kDa)

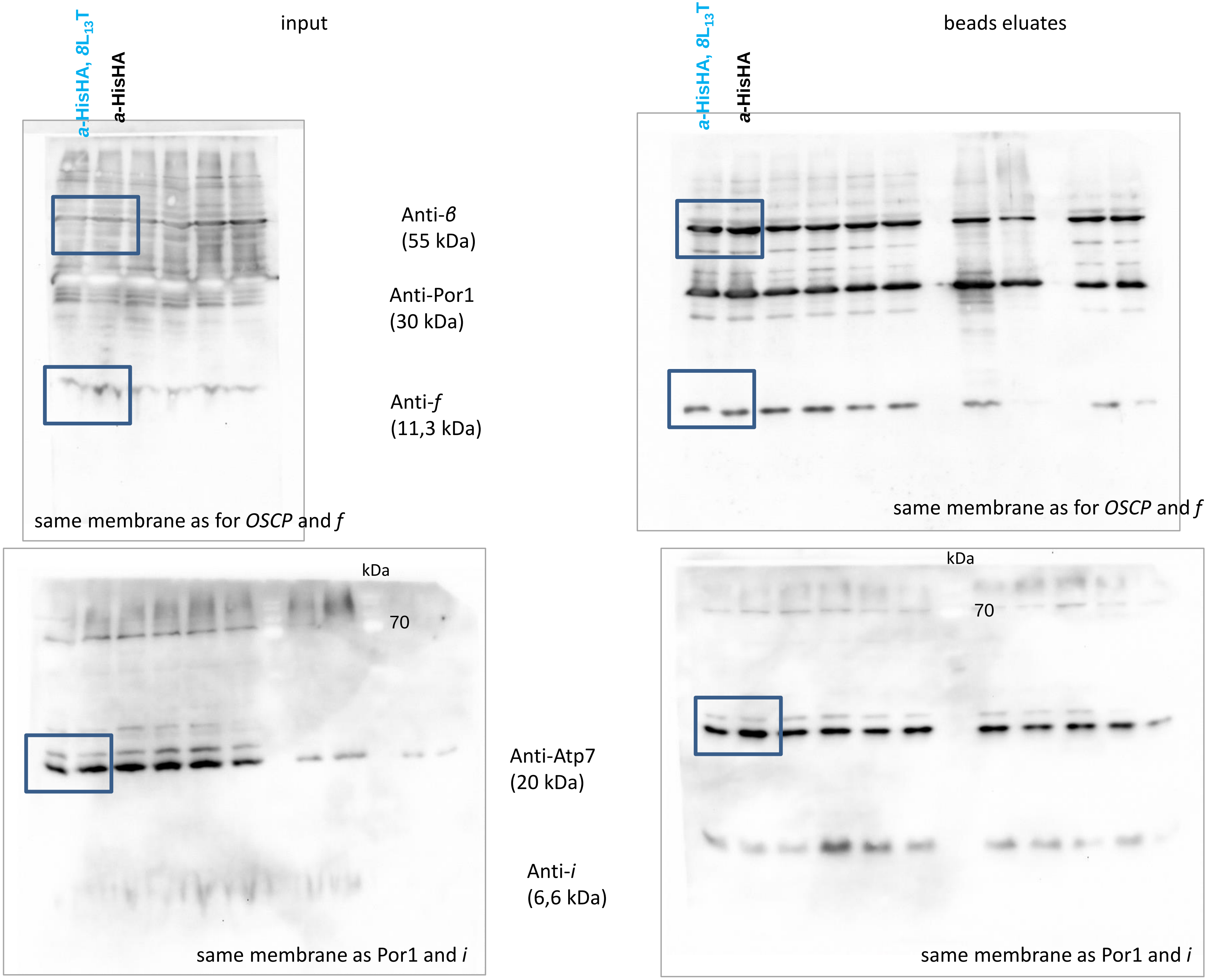

input
beads eluates
a-HisHA, 8L13T
a-HisHA
a-HisHA, 8L13T
a-HisHA
Anti-β (55 kDa)
Anti-Por1 (30 kDa)
Anti-f (11,3 kDa)
same membrane as for OSCP and f
same membrane as for OSCP and f
kDa
70
kDa
70
Anti-Atp7 (20 kDa)
Anti-i (6,6 kDa)
same membrane as Por1 and i
same membrane as Por1 and i

input

beads eluates

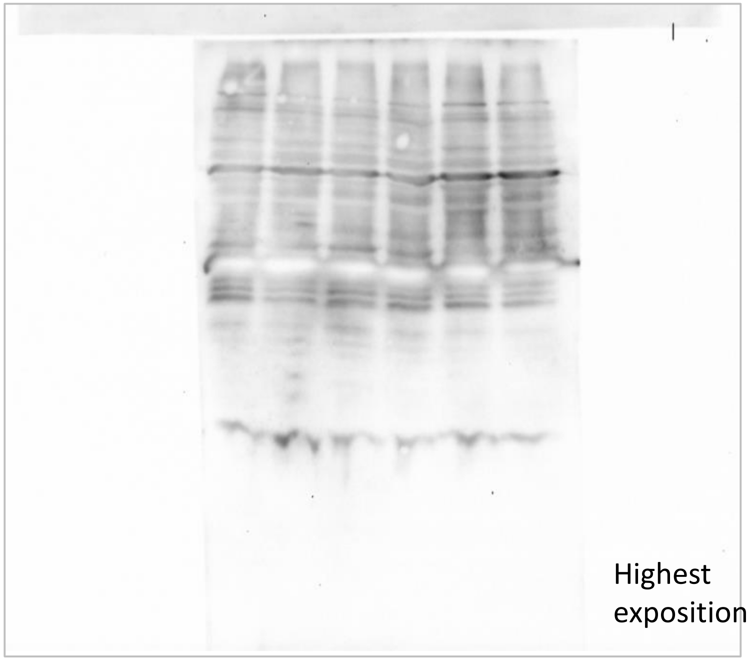

Highest exposition

Anti-β (55 kDa)

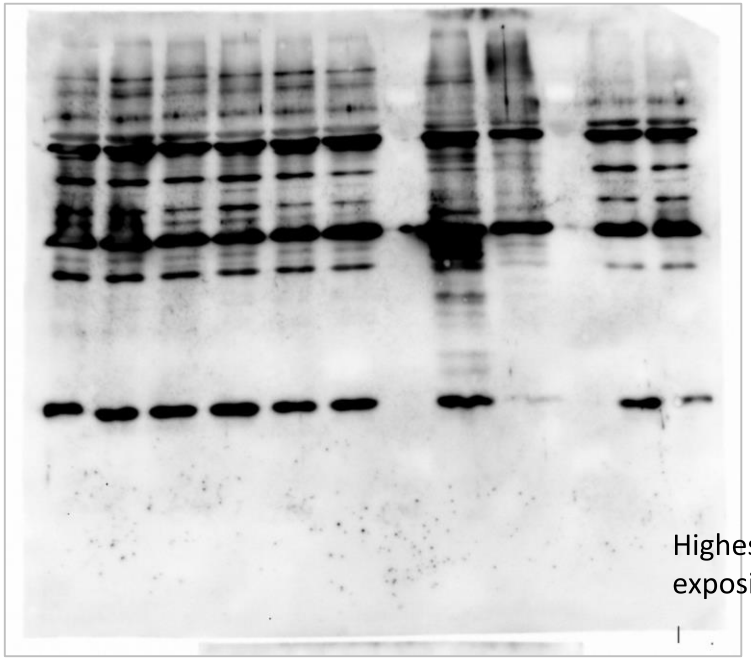

Highest exposition

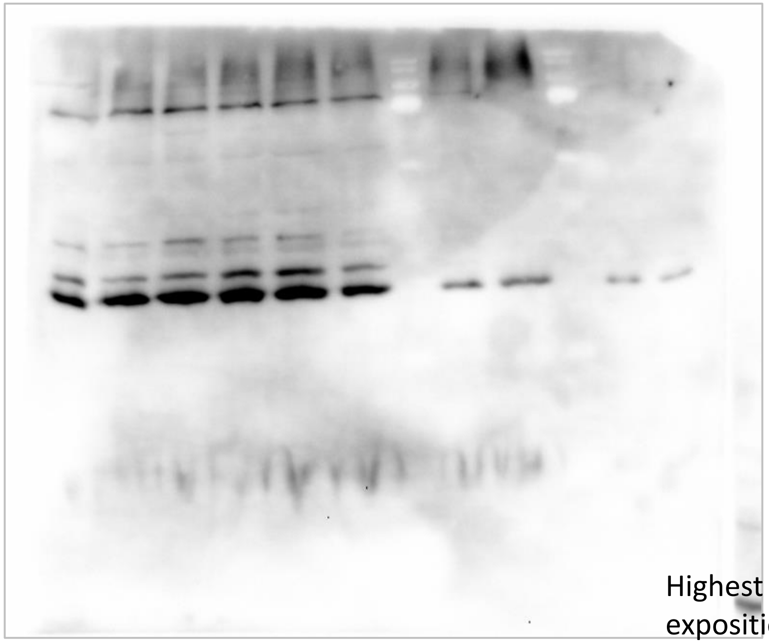

Highest exposition

Anti-Atp7 (20 kDa)

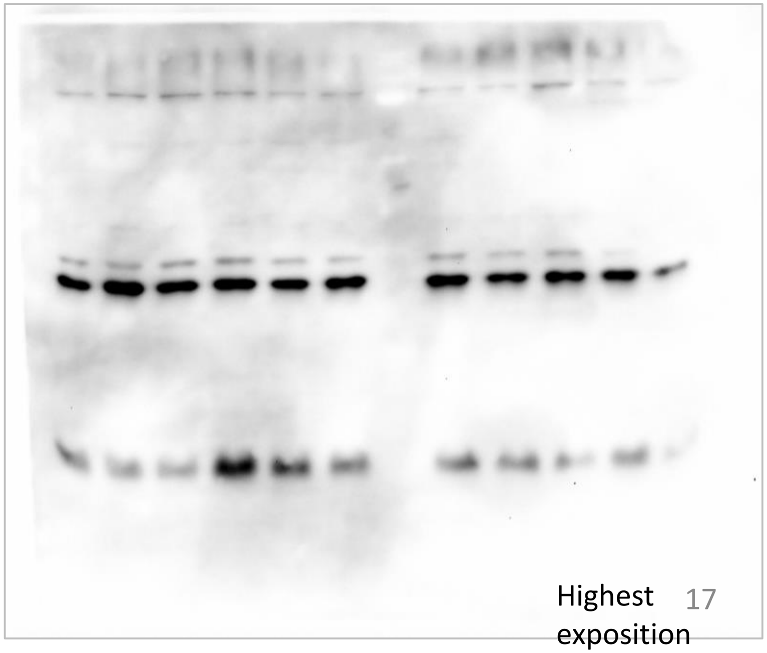

Highest exposition
